# *Streptococcus pneumoniae* Cell-Wall-Localized Phosphoenolpyruvate Protein Phosphotransferase Can Function as an Adhesin: Identification of Its Host Target Molecules and Evaluation of Its Potential as a Vaccine

**DOI:** 10.1371/journal.pone.0150320

**Published:** 2016-03-18

**Authors:** Yaffa Mizrachi Nebenzahl, Karin Blau, Tatyana Kushnir, Marilou Shagan, Maxim Portnoi, Aviad Cohen, Shalhevet Azriel, Itai Malka, Asad Adawi, Daniel Kafka, Shahar Dotan, Gali Guterman, Shany Troib, Tali Fishilevich, Jonathan M Gershoni, Alex Braiman, Andrea M Mitchell, Timothy J Mitchell, Nurith Porat, Inna Goliand, Vered Chalifa Caspi, Edwin Swiatlo, Michael Tal, Ronald Ellis, Natalie Elia, Ron Dagan

**Affiliations:** 1 Pediatric Infectious Disease Unit, Soroka University Medical Center, Beer Sheva, Israel; 2 The Shraga Segal Department of Microbiology, Immunology and Genetics, Faculty of Health Sciences, Ben-Gurion University of the Negev, Beer-Sheva, Israel; 3 The Faculty of Health Sciences, Ben-Gurion University of the Negev, Beer-Sheva, Israel; 4 NasVax Ltd, Ness Ziona, Israel; 5 Department of Cell Research and Immunology, Tel-Aviv University, Tel-Aviv, Israel; 6 Institute of Microbiology and Infection, College of Medical and Dental Sciences, University of Birmingham, Birmingham, United Kingdom; 7 National Institute of Biotechnology in the Negev, Ben-Gurion University of the Negev, Beer Sheva, Israel; 8 Department of Life Sciences, Ben-Gurion University of the Negev, Beer-Sheva, Israel; 9 Division of Infectious Diseases, University of Mississippi Medical Center, Jackson, MS, United States of America; ContraFect Corporation, UNITED STATES

## Abstract

In *Streptococcus pneumonia*, phosphoenolpyruvate protein phosphotransferase (PtsA) is an intracellular protein of the monosaccharide phosphotransferase systems. Biochemical and immunostaining methods were applied to show that PtsA also localizes to the bacterial cell-wall. Thus, it was suspected that PtsA has functions other than its main cytoplasmic enzymatic role. Indeed, recombinant PtsA and anti-rPtsA antiserum were shown to inhibit adhesion of *S*. *pneumoniae* to cultured human lung adenocarcinoma A549 cells. Screening of a combinatorial peptide library expressed in a filamentous phage with rPtsA identified epitopes that were capable of inhibiting *S*. *pneumoniae* adhesion to A549 cells. The insert peptides in the phages were sequenced, and homologous sequences were found in human BMPER, multimerin1, protocadherin19, integrinβ4, epsin1 and collagen type VIIα1 proteins, all of which can be found in A549 cells except the latter. Six peptides, synthesized according to the homologous sequences in the human proteins, specifically bound rPtsA in the micromolar range and significantly inhibited pneumococcal adhesion *in vitro* to lung- and tracheal-derived cell lines. In addition, the tested peptides inhibited lung colonization after intranasal inoculation of mice with *S*. *pneumoniae*. Immunization with rPtsA protected the mice against a sublethal intranasal and a lethal intravenous pneumococcal challenge. In addition, mouse anti rPtsA antiserum reduced bacterial virulence in the intravenous inoculation mouse model. These findings showed that the surface-localized PtsA functions as an adhesin, PtsA binding peptides derived from its putative target molecules can be considered for future development of therapeutics, and rPtsA should be regarded as a candidate for vaccine development.

## Introduction

*Streptococcus pneumoniae* (pneumococcus) colonizes the human nasopharynx asymptomatically and may therefore spread through the population. The asymptomatic colonization and the rapid spread of the bacteria are in themselves not a major health hazard, but as the result of the appearance of a virulent *S*. *pneumoniae* strain or of co-infection with another pathogen, *S*. *pneumoniae* can cause otitis media, pneumonia, bacteremia, meningitis and sepsis. In view of the serious consequences of *S*. *pneumoniae* infection and the increasing antibiotic resistance of this pathogen, there is a pressing need for safe and effective therapeutic approaches and for preventive vaccines [1]. One of the currently used vaccines is based on 23 selected capsular polysaccharides [2]. This vaccine has been shown to be 60% effective in preventing invasive pneumococcal diseases in the elderly [3] but does not elicit long-term immune memory or protective immune responses in children under two years of age [4, 5]. To address this problem, pneumococcal capsular polysaccharides have been conjugated to various carrier proteins [6] to produce the so-called conjugate vaccines. These vaccines, which currently include 10–13 serotypes, do induce immune memory and a protective immune response in infants. However, they do not give complete protection in that the pneumococcal serotypes not included in these conjugate vaccines are associated with carriage and disease [7, 8]. Thus, new therapeutic approaches and improved vaccines are being sought among newly identified bacterial virulence factors.

Among the molecules known to initiate the *S*. *pneumoniae*-host interaction that leads to nasopharyngeal colonization are those making up the two types of pneumococcal pilus. The first type, an oligomeric appendage known as the type I pilus, is encoded by the *rlrA* operon [9], and the second is encoded by the pilus islet known as PI-2 [10]. A recent publication revealed that the RrgA protein of the type I pilus binds to toll-like receptor (TLR) 2 and is thus an inflammatory and adherence-promoting structure [11].

Following the initial attachment of the bacterium, the bacterial capsule is shed, thereby providing the bacterium with access to the respiratory mucosa and facilitating the exposure of the adhesins that are embedded in the bacterial cell-wall or the cytoplasmic membrane [12]. Among the *S*. *pneumoniae* cell-wall and membrane adhesins are the lipoprotein PsaA [13] and the Pav-A protein [14]. PsaA binds to the E-cadherin receptor [15], while Pav-A binds to the extracellular matrix (ECM) protein fibronectin, which, in turn, binds to the integrin receptor [14]. Other *S*. *pneumoniae* adhesins are fructose bisphosphate aldolase, which binds the flamingo cadherin receptor [16], and NADH oxidase (NOX), which binds the ECM protein laminin α5, among other putative receptors [17].

Following attachment, *S*. *pneumoniae* can invade the mucosal cells via binding of either the bacterial phosphorylcholine to the platelet-activating factor receptor (PAF-R) [18] or of the choline-binding protein A (CbpA also known as SpsA or PspC) [19] to the polymeric immunoglobulin receptor (pIgR) or to secretory IgA. The adhesins phosphorylcholine and CbpA are considered to be invasins, since they facilitate transcytosis through the mucosal epithelial cell layer. PAF-R is present in both epithelial and endothelial cells, and pneumococcal binding may initiate the PAF-R recycling pathway, which transports the bacteria to the basal membrane of the host and leads to the development of an invasive disease. Similarly, following attachment to pIgR, the pneumococci exploit the pIgR recycling pathway to traverse the epithelium from the apical to the basal membrane [18, 20, 21]. It should be noted that many adhesins and invasins, such as PspA, CbpA, PavA, PavB, and PhtD, are known to be immunogenic and to elicit a protective immune response in mouse model systems [22–25]. Moreover, PhtD has recently been shown to elicit an immune response in phase I/II clinical trials [26, 27].

It is thus apparent that a significant body of knowledge has accumulated on the pneumococcal classical surface proteins with known export and cell-wall anchorage sequences, namely, choline-binding proteins, LPxTG-carrying proteins, and lipoproteins. In contrast, knowledge about the mechanisms of export or anchoring of the pneumococcal non-classical surface-associated proteins is limited [28–30]. It is, however, known that these proteins frequently have more than one function or activity in a cell at different anatomical locations—the cytoplasm and the cell wall. Many of the non-classical cell-wall proteins are hence moonlighting proteins that function as adhesins in the cell-wall and in other roles in the cytoplasm. We propose that one such non-classical cell-wall-associated protein is phosphoenolpyruvate protein phosphotransferase (PtsA), a housekeeping protein, with neither a signal sequence nor a choline binding domain that is highly conserved within the sequenced *S*. *pneumoniae* strains. In the cytoplasm, PtsA, a prokaryotic protein, functions as the first enzyme (EI) in the phosphotransferase systems (PTSs) that are responsible for the internalization and concurrent phosphorylation of monosaccharides. PtsA transfers a phosphate group from phosphoenolpyruvate (PEP) via HPr to a histidine in the next enzyme (EII) in this pathway [31]. This phosphate is then sequentially transferred to the monosaccharide-specific membrane-spanning proteins responsible for the phosphorylation and internalization of the monosaccharides.

In the current study, we show that PtsA is present on the surface of the pneumococcus, where it functions as an adhesin. A search for putative PtsA target molecules did indeed identify such molecules, and their existence was confirmed in host target cells. Short peptides, derived from PtsA putative target molecules, were identified and shown to interfere *in vitro* with bacterial adhesion to lung- and pharyngeal-derived cell lines. Moreover, it was also shown that these putative target peptides interfered with bacterial nasopharynx and lung colonization *in vivo* in a mouse model system. Finally, the ability of rPtsA to elicit a protective immune response in mice against *S*. *pneumoniae* inoculation was demonstrated.

## Materials and Methods

### Ethics Statement

All human studies, protocol revisions, and consent procedures were approved by the Helsinki Ethics Committee of the Soroka University Medical Center, Beer Sheva, Israel (Permit number: 10391). Written informed consent was obtained from the next of kin, carer or guardian of the minor/child participants involved in this study.

Experiments involving animals were carried out in strict accordance with the recommendations in the Guide for the Care and Use of Laboratory Animals of the National Institutes of Health. The protocol was approved either by the Institutional Animal Care and Use Committee of the Ben-Gurion University of the Negev, Beer Sheva, Israel (Permit number: 53.08.08) or by the University of Mississippi Medical Center, in accordance with the guidelines and approval of its IACUC (permit number: 00163).

### Immunization and Inoculation of Mice

Seven-week-old BALB/cOlaHsd (BALB/c) female mice (Harlan Laboratories, Israel) or seven-week-old CBA/CaHN-*Btk*^*xid*^ (CBA/N^*xid*^; Jackson Laboratories, Bar Harbor, ME, USA) mice were housed in sterile conditions under 12-h light/dark cycles and fed Purina Chow and tap water *ad libitum*.

BALB/c (highly resistant to *S*. *pneumonia*) or CBA/N^*xid*^ (highly susceptible to *S*. *pneumoniae*) mice strains were immunized subcutaneously (SC) with 25 μg of rPtsA emulsified with complete Freund’s adjuvant (CFA) and subsequently boosted (days 14 and 28) with incomplete Freund’s adjuvant (IFA). On day 42, the BALB/c and CBA/N^*xid*^ mice were challenged intranasally (IN), under deep anesthesia using Isoflurane (Piramal Critical Care Inc., PA, USA), with a sublethal dose of strain WU2 (5 × 10^7^ and 5 ×x 10^5^, respectively). Mice were humanely sacrificed 3 and 24 h later by CO_2_ asphyxiation, as recommended by the AVMA Guidelines for Euthanasia in Animals: 2013 Edition (https://www.avma.org/KB/Policies/Documents/euthanasia.pdf). Strain WU2 cells were pretreated with the respective peptides and then inoculated IN into adult CBA/N^*xid*^ mice. Mice were euthanized 3, 24 or 48 h later with CO_2_. The nasopharynx and right lobe lung were excised, homogenized and plated onto blood agar plates for bacterial colony counting. For survival experiments, after an immunization regime similar to that described above, BALB/c or CBA/N^*xid*^ mice were challenged with a lethal dose of strain WU2 either IN (10^8^ CFU and 10^6^ CFU, respectively) or intravenously (10^4^ CFU). In these survival experiments, the mice were humanely euthanized by CO_2_ asphyxiation if they become became moribund or showed evidence of distress. The following criteria were considered sufficient evidence of distress to warrant such intervention in order to minimize pain and suffering of the animals: severe weight loss (20% body weight); reluctance or inability to move freely; appearance of bristle fur; social disengagement; refusal or inability to eat or drink. No analgesic treatment was provided, as such treatment may alter the immune response and may independently affect the outcome of the experiments [32].

### Reagents

Unless otherwise stated, all chemicals and biochemical materials were of the highest purity available and were purchased from Sigma-Aldrich (St. Louis, MS, USA).

### Bacterial Strains and Growth Conditions

Two genetically unrelated encapsulated *S*. *pneumoniae* strains, serotype 2 strain D39 [33] and serotype 3 strain WU2 [34], and their unencapsulated derivatives, strain R6 (ATCC, Rockville MD) and strain 3.8DW [35], respectively, were used. In addition, in this study we used also strains 1, 5, 6B, 9V, 14R. 23F (1), and 14DW (kindly provided by Prof. David Watson, Dallas USA). Pneumococci were grown in Todd-Hewitt broth supplemented with 0.5% yeast extract (THY) or on blood agar plates, as previously described [36]. Two *Escherichia coli* strains were used as cloning vectors, DH5α UltraMAX (DH5α; Invitrogen Corp, Carlsbad, CA, USA) and BL21 (DE3) pLysS (BL21; Promega Corp, Madison, WI, USA) and were grown in lysogenic broth (LB).

### Enrichment of *S*. *pneumoniae* Total Cell-Wall and Membrane Proteins

Isolation of the cell-wall proteins was performed as previously described with minor modifications [37]. Briefly, bacteria, grown to mid log (OD_629_ 0.5), were pelleted by centrifugation. The supernatant was discarded, and bacteria were resuspended in protoplast buffer (20% sucrose, 2.5 mM MgCl_2_, 5 mM Tris-Cl, pH 7.4 and mixed protease inhibitors) and incubated with mutanolysin (200 U/ml) at 37°C for 1 h to release the cell-wall proteins. Following centrifugation, supernatants containing *S*. *pneumoniae* total cell-wall proteins were collected and stored at –20°C for further processing. The pellet was resuspended in the protoplast buffer and sonicated at 20,000 Hz for 15 s. Following centrifugation at 25,000 RPM for 45 min, the membrane pellet was dissolved in protoplast buffer containing 0.5% Triton X-100 and centrifuged at 40,000 RPM for 60 min. The supernatant containing the membrane proteins was collected and stored at -20°C. The cytoplasmic fraction was obtained following sonication of the bacteria at 20,000 Hz for 15 s. Following centrifugation at 25,000 RPM for 45 min, the supernatant was collected and used as the cytoplasmic fraction.

### Fractionation of *S*. *pneumoniae* Cell-Wall Total Proteins into Cell-Wall Lectin and Non-Lectin Proteins

The cell-wall total proteins were fractionated into cell-wall non-lectin and cell-wall lectin proteins by using fetuin-agarose affinity chromatography, as previously described with minor modifications [38]. Briefly, cell-wall total proteins were loaded onto the column and washed with the protoplast buffer. The proteins that did not bind to the column were regarded as cell-wall non-lectin proteins and were frozen at -20°C. The proteins that bound to the column (cell-wall lectins) were eluted with 50 mM ammonium acetate, pH 3.5, dried in a vacuum centrifuge, re-suspended in PBS, and stored for further analysis at -20°C.

### Identification of PtsA

Separation of the non-lectin cell-wall fraction and 2D PAGE were performed as previously described [39]. Protein spots were excised from the gel, washed with 150 μl of washing solution (50% methanol and 5% acetic acid) for 3 h and dehydrated with 200 μl of acetonitrile (5 min). Reduction and alkylation were performed with 30 μl of 10 mM DTT and 30 μl of 100 mM iodoacetamide. After rehydration with 200 μl of 100 mM ammonium bicarbonate (10 min), gel pieces were dehydrated, completely dried in a vacuum centrifuge, and subjected to enzymatic cleavage (porcine trypsin; Promega) for 16 h at 37°C in 50 mM ammonium bicarbonate buffer. Extraction of the peptides was performed by adding 30 μl of 50 mM ammonium bicarbonate and 5% formic acid. Matrix-assisted laser desorption ionization-time of flight mass spectrometry (MALDI-TOF-MS) analysis was performed using a REFLEX III (Bruker) mass spectrometer. Peptide peak lists were searched against the database of *S*. *pneumoniae* TIGR4 (www.matrixscience.com).

### Cloning, Expression and Purification of Recombinant Proteins

The nucleotide sequence of the locus SP_RS05795, coding for the WP_000138135 protein was amplified from pneumococcal serotype 3 strain WU2 genomic DNA according to the published sequence of serotype 4 strain TIGR4 by PCR with the following primers: **Forward**: 5'-GGATCCATGACAGAAATGCTTAAAG-3' and **Reverse:** 5'-GAGCTCTTAATCAAAATTAACGTATTC-3' supplemented with restriction enzyme sequences of *Bam*HI on the 3' end and *Sac*1 on the 5' end (Takara Biomedicals, Otsoshiga, Japan). The amplified product was cloned into the pHAT expression vector (BD Biosciences Clontech, Palo Alto, CA, USA) and transformed into *E*. *coli* DH5a UltraMAX ultracompetent *E*. *coli* cells. Insert DNA was analyzed by PCR in ampicillin-resistant transformants (data not shown). Verification of sequence identity was performed by plasmid insert sequencing (data not shown). The vector was purified using the Qiagen High-Speed Plasmid Maxi Kit and transformed into *E*. *coli* host expression strain BL21(DE3)pLysS (Invitrogen). Bacteria were grown overnight, and expression of recombinant rPtsA was induced by use of 1 mmol/L isopropyl-β-d-thiogalactopyranoside for 5 h. The cells were harvested and lysed, and the protein was purified under native conditions and then dialyzed against PBS for imidazole removal. Separation of the tagged-purified protein on SDS-PAGE showed that the 75-kDa rHAT-PtsA fusion protein was ~95% pure (S1A Fig). Immunoblotting with anti-HAT antiserum further confirmed the identity of the recombinant protein (S1B Fig). The discrepancy between the expected molecular weight of the fusion protein of 67.5 kDa (rPtsA 65.4 kDa plus 2 kDa HAT tag) may result from proteolysis. Alternatively, the altered electrophoretic mobility may stem from possible lipid moiety attachment to the protein, since TMpred software predicted the existence of hydrophobic regions in PtsA amino acids 517–535 to be a transmembrane helix (with a score of 525; http://www.ch.embnet.org/software/TMPRED_form.html) (S1B and S1C Fig). MALDI-TOF analysis of a peptide digest of this protein band identified rPtsA in 99% accordance with the expected PtsA protein (PI = 4.6, Mascot score = 92, Z score = 2.43, extent of sequence coverage = 39%).

**Fig 1:**
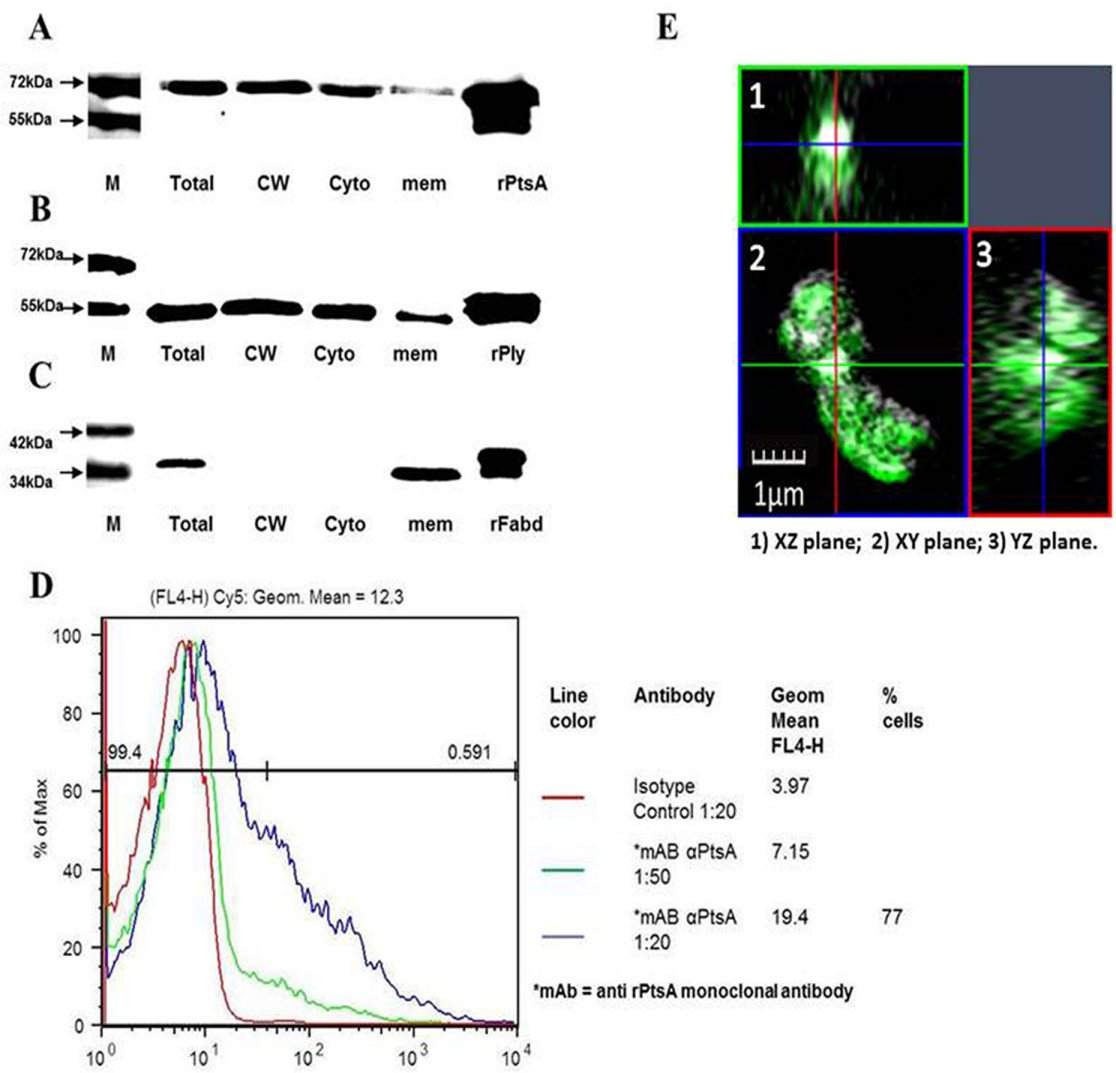
Surface expression of PtsA. A) Immunoblot of untagged rPtsA: total *S*. *pneumoniae* extract (Total), total cell-wall proteins (CW), cytoplasmic proteins (Cyto) and membrane extract (mem) were probed with rabbit anti-rPtsA antiserum. B) Immunoblot of recombinant pneumolysin (rPly): total *S*. *pneumoniae* extract, total cell-wall proteins, cytoplasmic proteins and membrane extract were probed with rabbit anti-rPly antiserum. C) Immunoblot of tagged rFabD: total *S*. *pneumoniae* extract, total cell-wall proteins, cytoplasmic proteins and membrane extract were probed with rabbit anti-rFabD antiserum. D) FACS analysis of PtsA surface expression using anti-rPtsA monoclonal antibody (mAb) and isotype control IgG. Red line–preimmune control serum; green line–anti rPtsA mAb diluted 1:50; blue line–anti rPtsA mAb diluted 1:20. M = molecular weight markers. E) SIM orthogonal view image demonstrating the surface staining with mouse anti rPtsA antibody detected with donkey anti-mouse IgG (green). Three planes XZ, XY and YZ are shown. PtsA is visible on the perimeter of the bacterium in green, surrounding the white bacterial genome.

The gene for production of the untagged rPtsA was amplified from TIGR 4 strain (using the same primers as above) and cloned into pET30a+. *E*. *coli* xl1 (Agilent Technologies, Santa Clara, CA) was transformed with pET30a+^*ptsa*^. *E*. *coli* were grown and the plasmid was isolated and used to transform *E*. *coli* BL21. The *E*. *coli* BL21 cells were grown, centrifuged, resuspended in 10 mM Tris-HCl/10 mM EDTA (pH 8) with PMSF (final concentration 0.1 mg/ml), and then treated with lysozyme and DNase. This procedure was followed by sonication, centrifugation and precipitation of the protein using ammonium sulfate. The fraction at 70% saturation was solubilized with 10 mM Tris-HCl PMSF. The protein was purified ×3 by fractionation on Superdex 200s. Following removal of lipoprotein polysaccharide on a Detoxi-Gel^TM^ Endotoxin Removing Gel column (Pierce), the pooled protein was concentrated to 1 mg ml^-1^, dialyzed against NaHCO_3,_ and lyophilized. The rPtsA was resuspended at 1 mg ml^-1^ and separated on 1D SDS PAGE. The rPtsA was found to run as a ~ 70–72 kDa protein (S1D Fig). An immunoblot of the untagged protein with rabbit anti-rPtsA antiserum confirmed that under these conditions rPtsA runs as a ~72 kDa protein (S1E Fig). MALDI TOF analysis detected two peaks, one at 63,932.49 and the other at 62,492.49 Da, which are in better agreement with the hypothetical molecular weight predicted by the NCBI databases (63,165Da). Moreover, a peak at 120,090.67 Da detected by the MALDI analysis may indicate dimerization of PtsA, which is its enzymatic active form (S1E Fig). Of note, the biological experiments were performed using the tagged-rPtsA. The immunoblot analysis in Fig 1A was performed with the untagged-rPtsA as positive control.

### Preparation of Rabbit Antiserum

Three-month-old white albino rabbits (Harlan Laboratories, Israel) were immunized intramuscularly (IM) with 200 μg HAT-rPtsA emulsified with CFA (1:1) in the first immunization or with IFA in booster immunizations. Two weeks after their final immunization, the rabbits were exsanguinated, and sera were prepared.

### Immunoblot Analysis

Cell-wall, membrane and cytoplasmic pneumococcal proteins, 40 μg each, were separated by SDS-PAGE under reducing conditions and transferred to nitrocellulose membranes (Bio-Rad, Carlsbad, CA, USA), as previously described [40]. The identity of PtsA was confirmed by immunoblot analysis using either rabbit or mouse anti-rPtsA antiserum. Membranes were developed using MicroChemi 4.2 (DNR, Israel), and thereafter the molecular weight markers were added to the chemiluminescent image (Markers overlay). As negative control either preimmune serum was used or the primary antibody was excluded (to reduce redundancy the negative blots are not presented).

### Flow Cytometry of *S*. *pneumoniae*

Anti-rPtsA Monoclonal antibodies (mAb) were obtained using available procedures. Briefly, BALB/c mice were immunized 4 times with rPtsA, followed by harvesting of splenocytes for fusion with NSO cells by standard techniques. The hybridomas were tested for reactivity with rPtsA by ELISA. Positive clones were further subcloned, and the clone with the highest reactivity to the protein was adapted to serum-free media. The cells were expanded, and the mAb was purified from the culture supernatant by Protein G affinity chromatography. The reactivity of the purified mAb was reconfirmed by ELISA (data not shown) before sorting by FACS. The purified mAb preparations were in the range of 0.6–1.3 mg/mL^-1^. Purified IgG from naive mice was used as the control. An additional control was the exclusion of the primary antibody (not shown).

Flow cytometry was performed as previously described [41]. Briefly, strain R6 bacteria were incubated with anti-rPtsA mAb or isotype control mouse serum, washed, and stained with Alexa Fluor 647^®^-conjugated goat-anti-mouse-IgG (Jackson ImmunoResearch, West Grove, PA). Flow cytometry was performed using a FACSCalibur flow cytometer (Becton Dickinson, Mountain View, CA), and data were acquired and analyzed using BD CellQuest^TM^ 3.3 software.

### Cell Lines

A549 cells (lung adenocarcinoma cells; ATCC, Rockville, MD, USA), which retain the morphological, biochemical and immunological characteristics of type II lung epithelial cells [42–44], and Detroit 562 cells (pharyngeal carcinoma derived cells; ATCC, Rockville, MD, USA) are extensively used in investigations of *S*. *pneumoniae* adhesion [45]. The cells were grown in DMEM supplemented with 10% fetal calf serum.

### Inhibition of Adhesion of Pneumococci to Cultured Human Cell Lines

**Fig 2:**
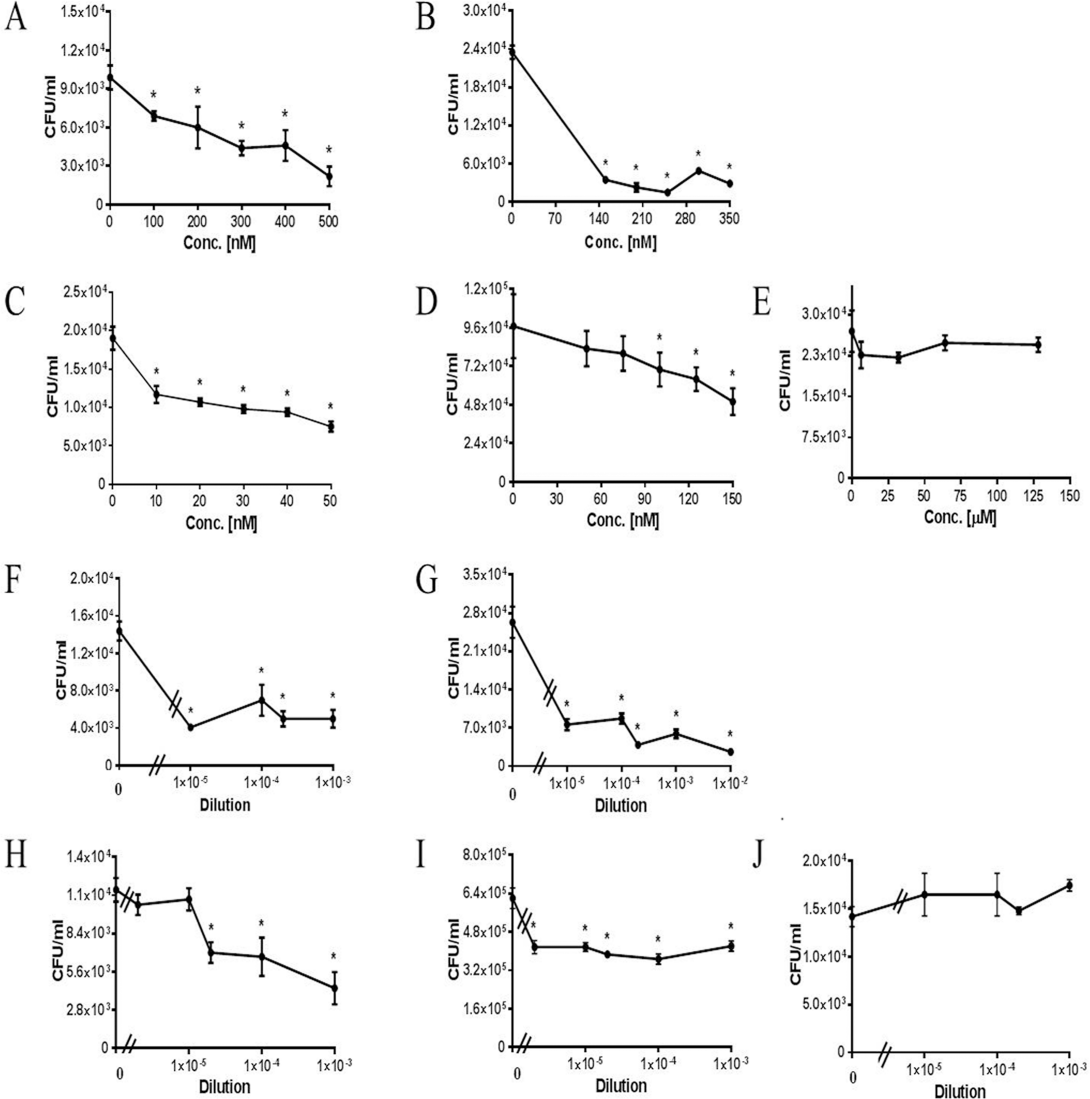
PtsA mediates *S*. *pneumoniae* adhesion to target host cells. **A–E.** A549 cells were grown to confluence and then blocked with 0,5% gelatin for 1 h. Excess gelatin was removed, and the A549 cells were incubated for 1 h with rPtsA at the denoted concentrations. Excess protein was then removed. *S*. *pneumoniae* was added for 1 h to the cells, non-adherent bacteria were removed, and cells were detached with trypsin and plated onto blood agar plates for counting. rPtsA inhibited the adhesion to A549 cells of: A) strain WU2 (p < 0.0001; r = −0.943), B) strain 3.8DW (p < 0.0001; r = −0.371), C) strain D39 (p < 0.0001; r = −1), and D) strain R6 (p < 0.0006; r = −1). E) rKLH, a protein used as a negative control, did not inhibit D39 adhesion to A549 cells (p = 0.8; r = −0.2). **F–J.** *S*. *pneumoniae* cells (WU2 and 3.8DW) were treated for 1 h with rabbit anti-rPtsA antiserum and added to gelatin-blocked A549 cells for 1 h; non-adherent bacteria were removed; and cells were detached with trypsin and plated onto blood agar plates for counting. Rabbit anti-rPtsA inhibited adhesion to A549 cells of: F) strain WU2 (p < 0.0001; r = −0.359); G) strain 3.8DW (p < 0.0001; r = −0.886); H) strain D39 (p < 0.0001; r = −0.943); and I) strain R6 (p < 0.0001, r = −0.406); J) Serum obtained from a rabbit prior to immunization did not inhibit the adhesion of strain WU2 to A549 (J p>0.005; r = +0.667). (p > 0.005; r = +0.667). Experiments were performed in triplicate and repeated at least 3 times. Values are means±SD. *Student's *t-*test p<0.05.

**Fig 3:**
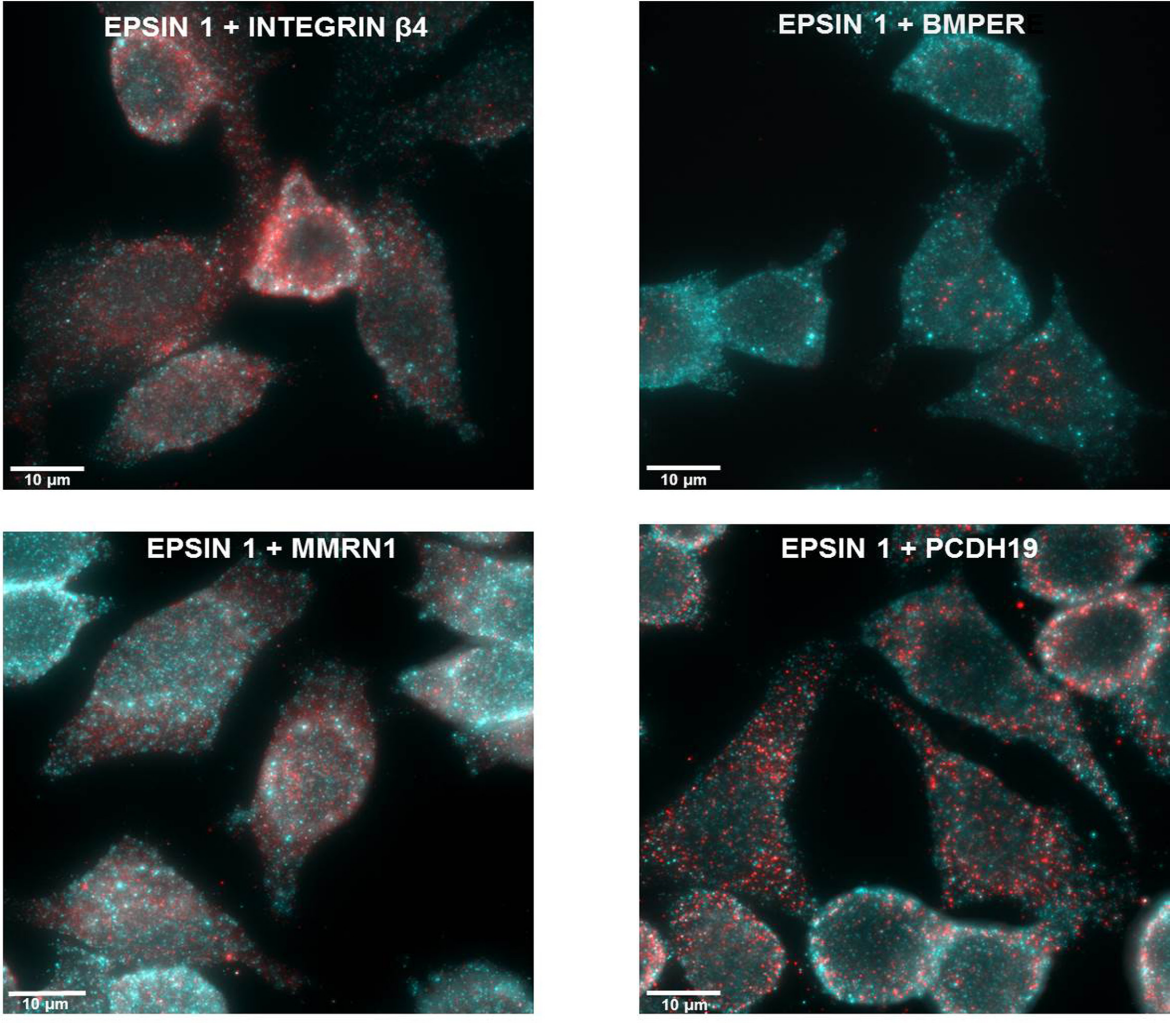
Putative target molecules in A549 cells. A549 cells were fixed with 4% para-formaldehyde and stained with a combination of a mouse anti Eps 1 and one of the following: rabbit anti Int β4, PCDH19, MMRN1, or BMPER. The secondary antibodies used were either Alexa Fluor 405-conjugated AffiniPure Goat Anti-Mouse IgG (blue) or Alexa Fluor 594-conjugated AffiniPure Goat Anti-Rabbit IgG (red) in accordance with the primary antibody used. 3D SIM images were taken using the Elyra SIM imaging system.

**Fig 4:**
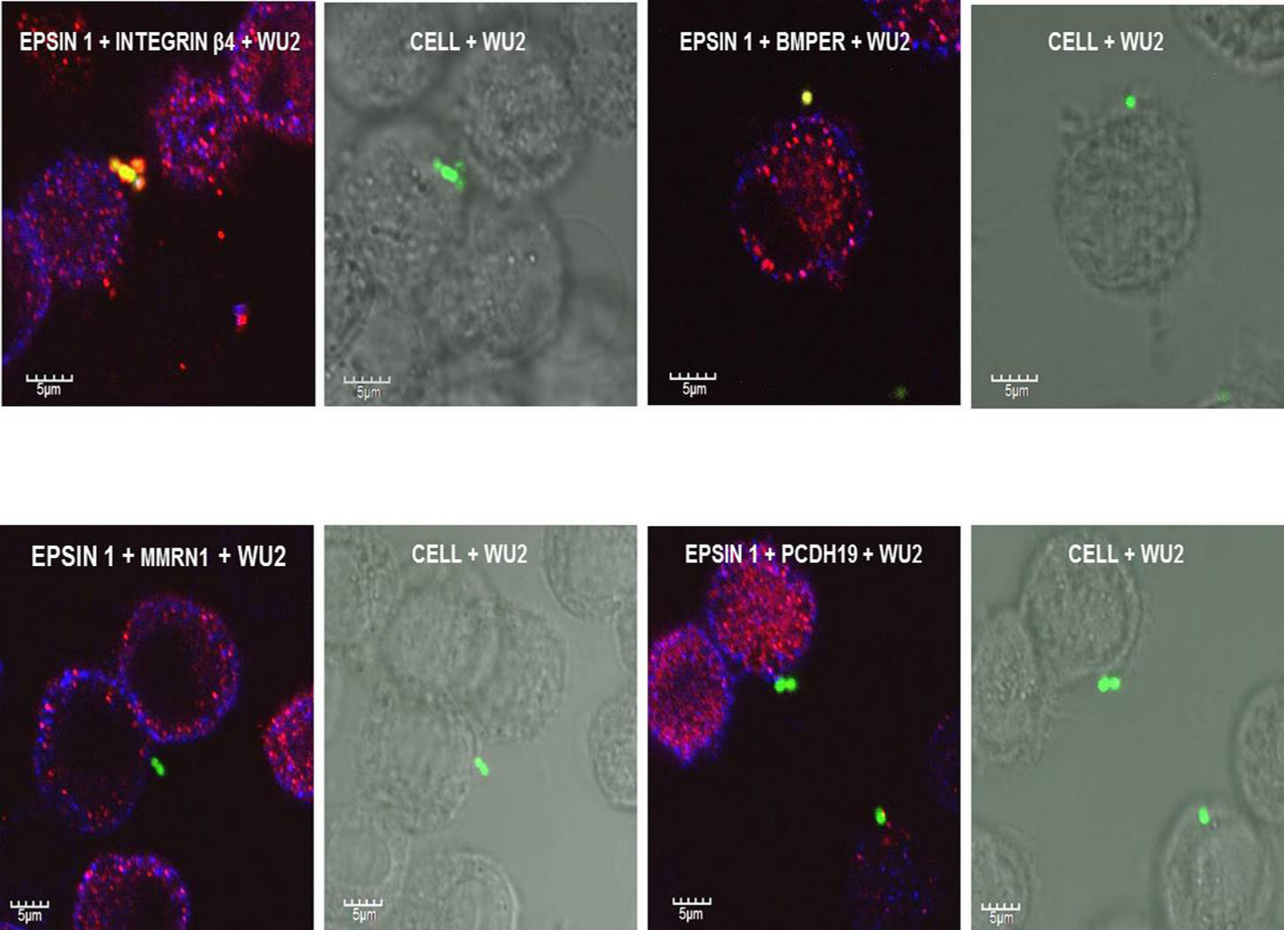
Immunostaining of *S*. *pneumoniae* adhesion to A549 cells. CFDA-stained *S*. *pneumoniae* strain WU2 cells were incubated with A549 cells for 1 h. Excess bacteria were removed, and the culture was fixed with 4% para-formaldehyde, stained, and viewed with a FluoView FV1000 confocal system (Olympus, Japan). The following stains were used: A) mouse anti-Eps 1 antiserum and rabbit anti-integrin β4 antiserum; C) mouse anti-epsin 1 antiserum and rabbit anti-BMPER antiserum; E) mouse anti-Eps 1 antiserum and rabbit anti-MMRN1 antiserum; and G) mouse anti-Eps 1 antiserum and anti-PCDH19 antiserum. The same cells viewed by Nomarsky microscopy overlaid with the CFDA-stained bacteria are shown, respectively, in B), D), F) and H). Secondary antibodies used were Alexa Fluor 405-conjugated AffiniPure Goat Anti-Mouse IgG (H+L) antiserum and Alexa Fluor 594-conjugated AffiniPure Goat Anti-Rabbit IgG (H+L (antiserum, in accordance with the primary antiserum used.

**Fig 5:**
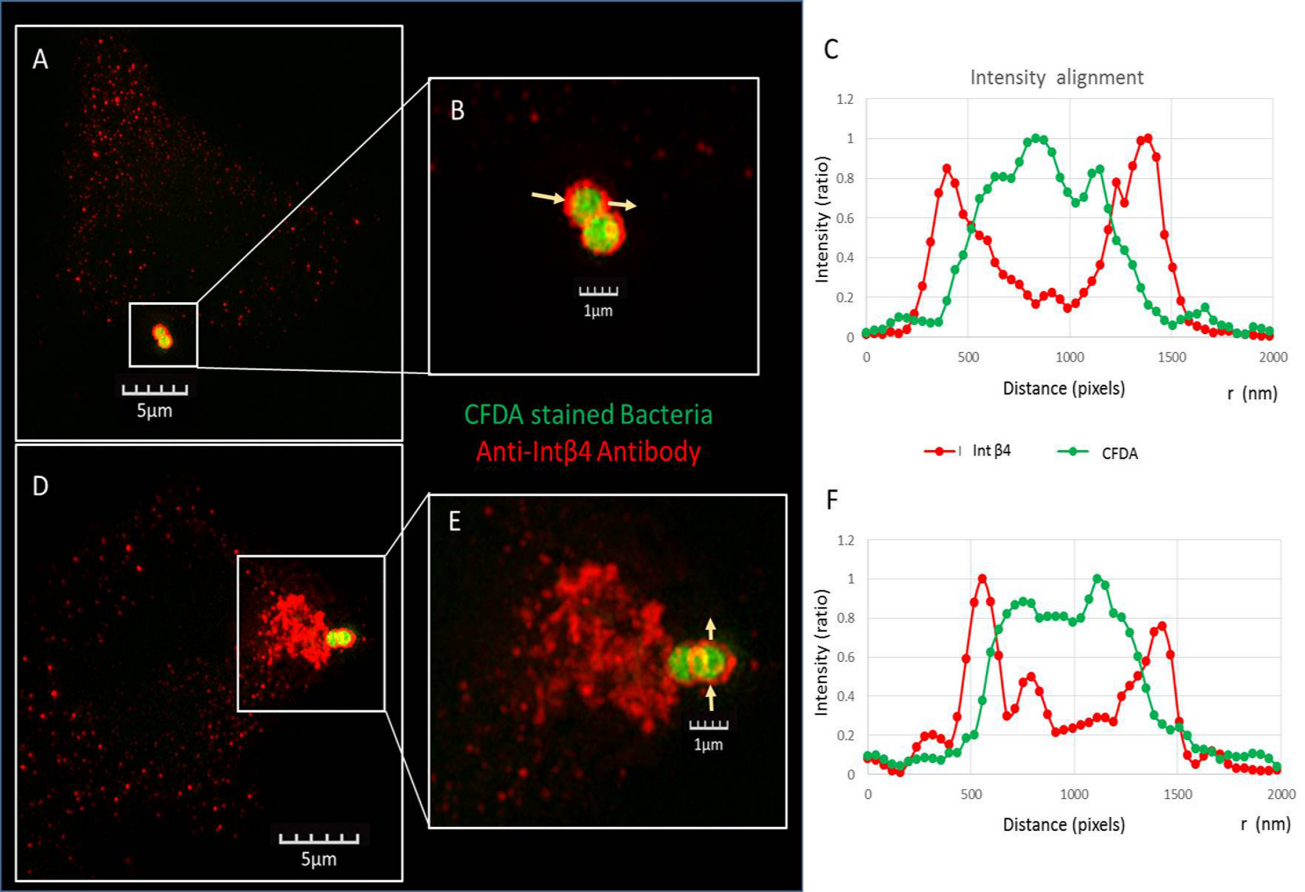
SIM images of CFDA-stained bacteria with Int β4 on A549 cells. CFDA-stained *S*. *pneumoniae* strain WU2 cells were incubated with A549 cells for 1 h. Excess bacteria were removed, and the culture was fixed with 4% para-formaldehyde and stained with a rabbit anti Int β4. Secondary antibody used was Alexa Fluor 594 (red) conjugated AffiniPure Goat Anti-Rabbit IgG (H+L). 3D SIM images were obtained with the Elyra SIM imaging system with a 63x oil objective (NA = 1.4); actual magnification of the image is as indicated in the scale bar. A) *S*. *pneumoniae* (CFDA 488 nm—green) adhering to A549 epithelial cells are seen coated by Int β4 (red). B) Higher magnification of bacteria (green) enveloped with Int β4 (red). C) Intensity alignment profile. D) Pedestal-like structure formed at the site of adherence of *S*. *pneumoniae* (green) to A549 cells recruits Int β4 (red). E) Higher magnification demonstrates the recruitment of Int β4 (red) to the pedestal-like structure underneath the adhered *S*. *pneumoniae* (green). F) Intensity alignment profile.

A549 and D562 cells were cultured on fibronectin-coated 96-well plates (Fig 2 and S2 Fig) or on Ibidi 8 well chambers (Ibidi GmbH, Planegg/Martinsried, Germany; Figs 3, 4 and 5) or on 96-well plates without fibronectin coating (Figs 7 and 8 and S3 Fig), as previously described [16]. Briefly, the cells were seeded at 5 × 10^4^ cells/well in DMEM supplemented with 10% fetal calf serum (FCS) (without antibiotics). Twenty four hours later, the cells (~10^5^ cells/well) were blocked with 0.5% gelatin for 1 h. rPtsA (0–500 nM) was then added to the cultured cells for 1 h of incubation. Excess protein was removed, and *S*. *pneumoniae* bacteria at a MOI of ~10:1 were added for additional 1 h of incubation. The inoculum size was verified in each experiment. Adhesion of the bacteria to fibronectin-coated plates, under our experimental conditions, did not exceed 2000 CFU/well, and this control was performed for each experiment. Following the incubation, excess bacteria were removed, and cells were detached with trypsin and plated onto blood agar plates for counting. All the inhibitors were used at concentrations below their toxic concentrations either to the bacteria or to the cells. Keyhole limpet hemocyanin (KLH) protein was used as a negative control. Experiments were conducted in triplicates and repeated on 3 different occasions.

**Fig 6:**
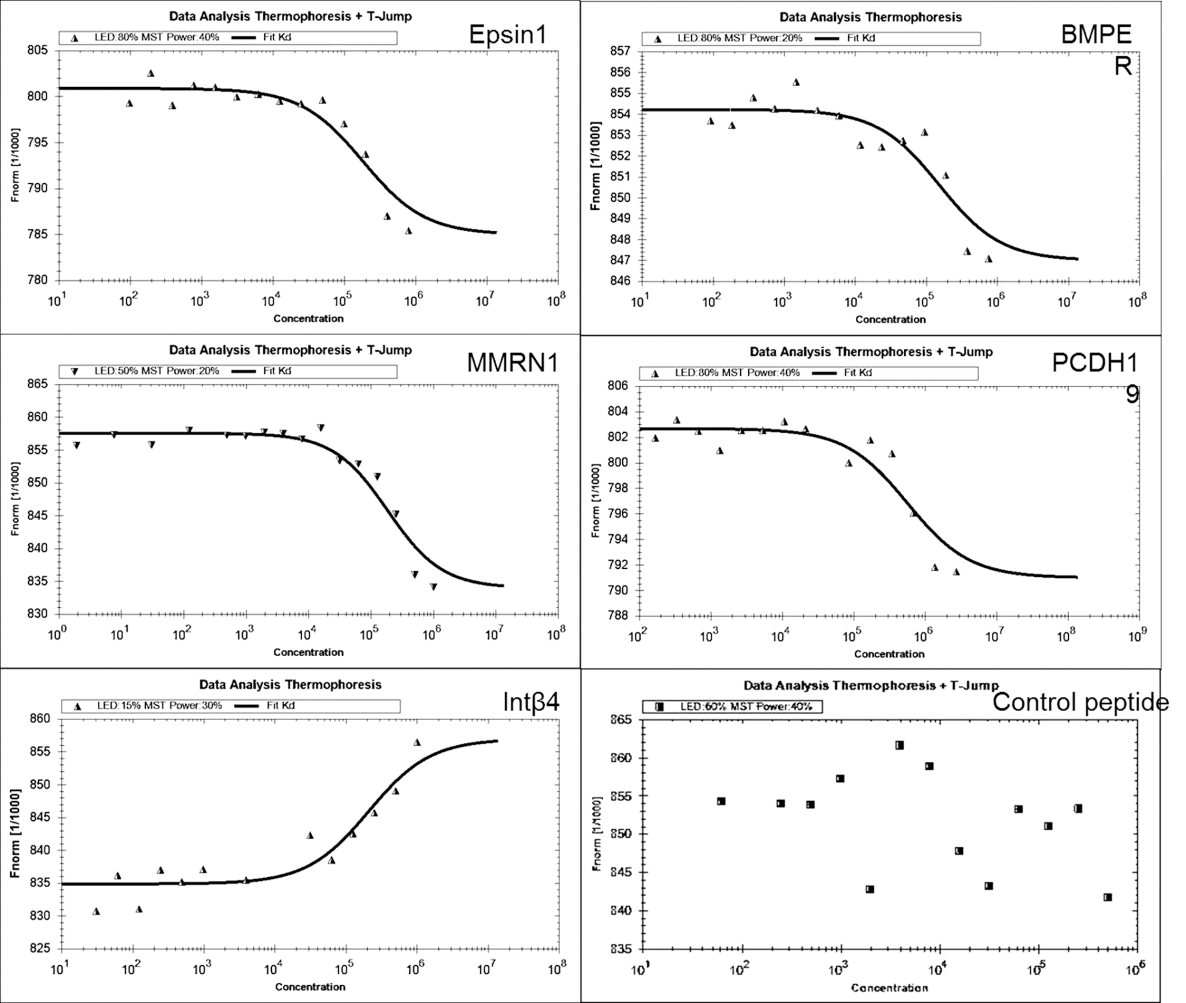
MicroScale thermophoresis (MST) analysis. In all cases, 16 serial dilutions of each putative receptor-derived peptide (~90 nM–3.175 mM) were mixed with a constant concentration of 335 nM labeled rPtsA protein. The normalized change in the fluorescence of the labeled rPtsA protein was plotted against the peptide concentration, and a fit was computed by the NTP program. The experiments were performed in triplicate. Presented here are the affinities of rPtsA binding to: the Eps1-derived peptide Kd = 43.900 ± 9.35 μM; the BMPER-derived peptide Kd = 21.00± 5.49μM; the MMRN1-derived peptide Kd = 391 ± 72.7 μM; the PCDH19-derived peptide Kd = 58.00 ± 13.4 μM; and the Int β4-derived peptide Kd = 115±20.5μM. For the control, namely, the Nox putative receptor (contactin 4)-derived peptide, a fit could not be generated due to a very low signal-to-noise ratio.

**Fig 7:**
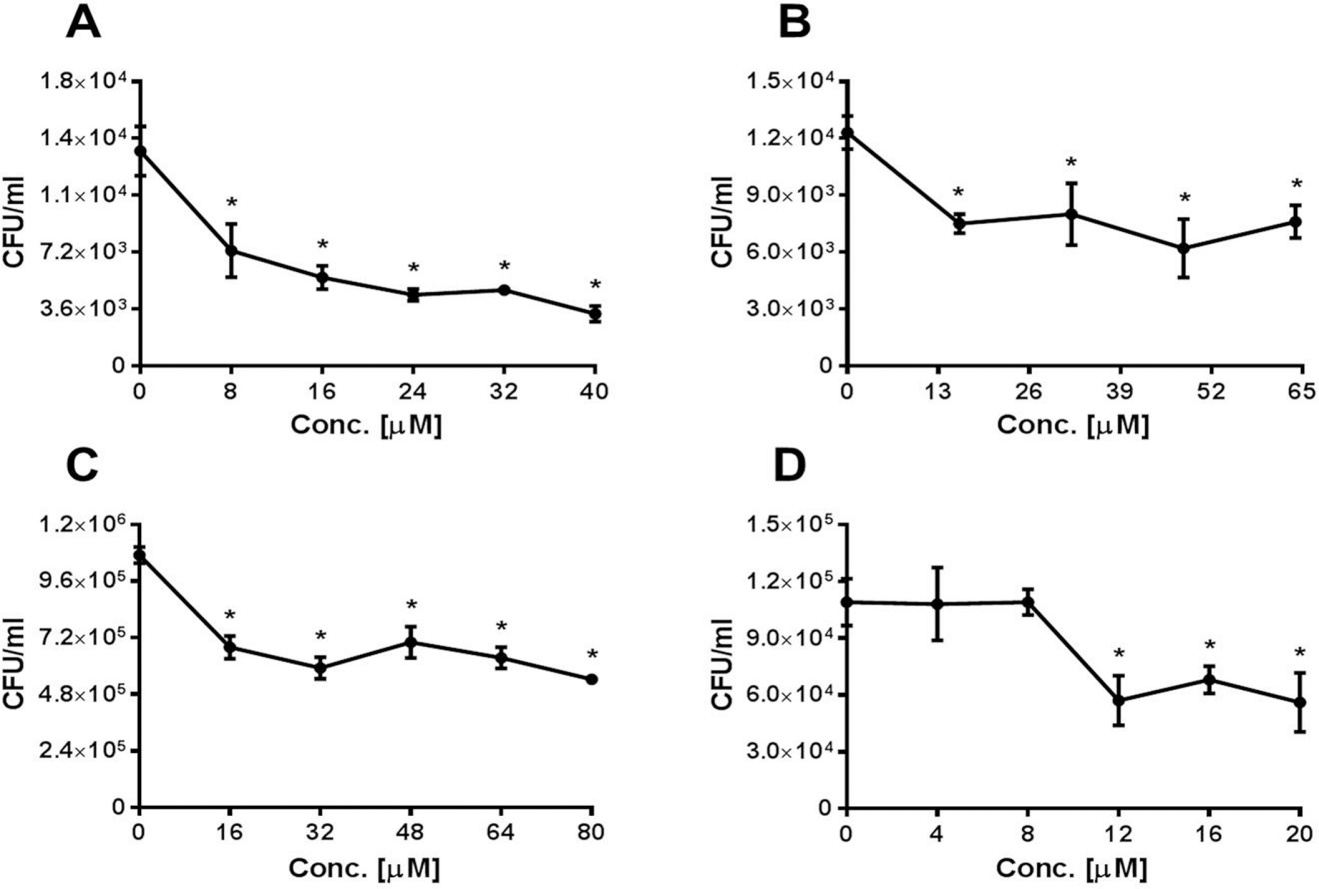
Inhibition of *S*. *pneumoniae* adhesion to A549 cells by Eps 1-derived peptide. *S*. *pneumoniae* cells were treated for 1 h with Eps 1-derived peptide and added to A549 cells for 1 h; non-adherent bacteria were removed; and cells were detached with trypsin and plated onto blood agar plates for counting. A) Strain WU2 (p < 0.0001; r = −0.943); B) Strain 3.8DW (p < 0.0001; r = −0.056); C) Strain D39 (p < 0.0001; r = −0.943); D) Strain R6 (p <0.0001; r = −0.406; *t-test p < 0.05). Experiments were performed in 3–6 replicates and repeated at least 3 times. Values are means±SD. *Student's *t-*test p < 0.05.

**Fig 8:**
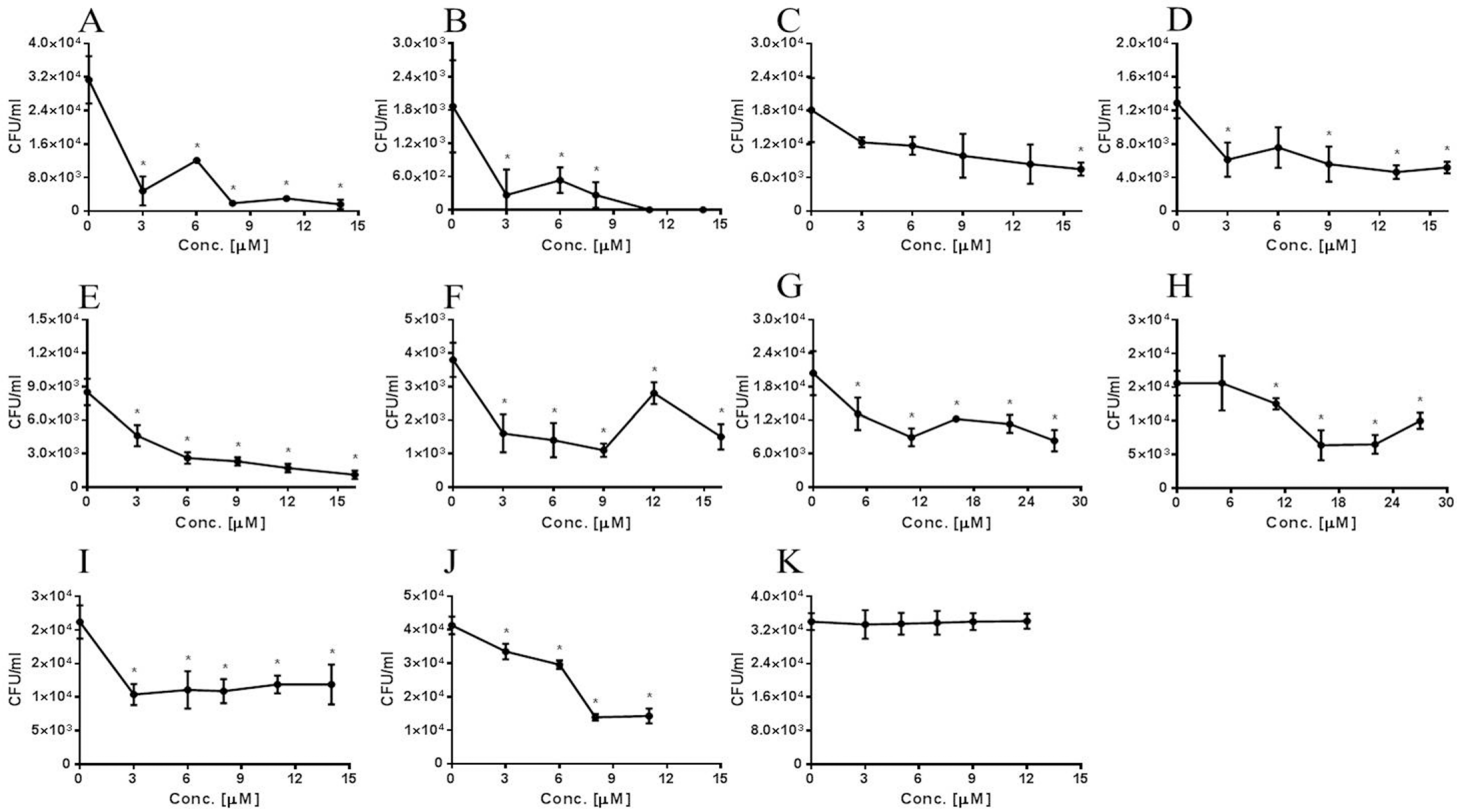
Inhibition of adhesion of *S*. *pneumoniae* cells to A549 cells by target-derived peptides. *S*. *pneumoniae* cells were treated for 1 h with peptide and added to A549 cells for 1 h; non-adherent bacteria were removed; and cells were detached with trypsin and plated onto blood agar plates for counting. A) BMPER, WU2 (*P* < 0.0001, r = −0.887); B) BMPER, 3.8DW (*P* < 0.0001, r = −883); C) Col VIIα WU2 (*P* < 0.0001, r = −1); D) Col VIIα, 3.8DW (*P* < 0.0001, r = −0.886); E) MMRN1, WU2 (*P* < 0.0001, r = −1); F) MMRN1, 3.8DW (*P* < 0.0001, r = −0.821); G) PCDH 19, WU2 (*P* < 0.0001, r = −0.829); H) PCDH 19, 3.8DW (*P* < 0.0001, r = −0.754); I) Int β4, WU2 (*P* < 0.0001, r = −0.5619); J) Int β4, 3.8DW (*P* < 0.0001, r = −0.967); K) No significant inhibition was observed with a scrambled peptide (K scrambled peptide, *P* = 0.9981). Experiments were performed in triplicates and repeated at least 3 times. Values are means±SD. *Student's *t-*test p<0.05.

Inhibition of adhesion to A549 cells by anti-rPtsA antiserum, phages or peptides was performed as previously described [16]. Briefly, bacteria were incubated for 1 h with rabbit anti-rPtsA antiserum, the phages, or the selected peptides. The bacteria were then added to the cultured human cells for an additional hour. Following this incubation, excess bacteria were removed, and the procedure was continued as described above. All the inhibitors were used at concentrations below their toxic concentrations either to the bacteria or to the cells. Negative controls included pre-immune serum, phage carrying no insert or a non-inhibitory insert, and a scrambled peptide. Each experiment was performed in triplicates and repeated on 3 different occasions and a representative experiment is presented.

### Immunofluorescence Microscopy

Immunostained images were obtained for live bacteria stained with polyclonal mouse anti-rPtsA antisera, detected with a Alexa-Flour 488-conjugated affinity pure donkey anti-mouse IgG (H+L) (Jackson ImmunoResearch Laboratories West Grove, PA, USA) The nuclei were stained with Hoёchst stain. Structured illumination microscopy (SIM) orthogonal view images were obtained using an Elyra SIM imaging system (Zeiss, Germany) with a 63× oil objective (NA = 1.4); actual magnification of the image is as indicated in the scale bar in the relevant figures. As negative control either preimmune serum was used or the primary antibody was excluded (the negative pictures are not presented).

A549 cells were cultured on Ibidi 8 well chambers (Ibidi GmbH, Planegg/Martinsried, Germany). The cells were fixed with 4% para-formaldehyde and stained with a combination of mouse anti-Eps 1 antiserum at 1:25 dilution and one of the following: rabbit anti-integrin β4 antiserum at 1:50 dilution (Santa Cruz Biotechnology, Inc. Delaware, CA, USA), anti-PCDH19 antiserum at 1:50 dilution, anti-MMRN1 antiserum at 1:200 dilution (Novus Biologicals, Littleton, CO, USA), or BMPER at 1:10 dilution (Abnova 9F, No. 108. Jhouzih, Taiwan). For bacterial adhesion studies, the combination of mouse anti- Intβ4 antiserum at 1:25 dilution and rabbit anti-Eps 1 at 1:50 dilution were used. Secondary antibodies used were either Alexa Fluor 405-conjugated AffiniPure goat anti-mouse IgG (H+L) antiserum at 1:125 dilution or Alexa Fluor 594-conjugated AffiniPure Goat Anti-Rabbit IgG (H+L) antiserum at 1:250 dilution (Molecular probes by Life Technologies, USA) in accordance with the primary antibody used. 3D images were acquired with either a FluoView FV1000 confocal system (Olympus, Japan) or an Elyra SIM imaging system (Zeiss, Germany). For SIM imaging, a 63× oil objective (NA = 1.4) was used. Five rotations and 5 phases were taken for each focal plane. Reconstructions were performed with Zen software (Zeiss, Germany). Shown here are maximum projections of 3D series. As negative control either preimmune serum was used or the primary antibody was excluded (to reduce redundancy the negative blots are not presented).

### CFDA Staining of the Bacteria

Bacteria were grown in THY broth to mid-late log phase as determined by OD. Aliquots of bacteria were harvested by centrifugation (13000 rpm), re-suspended in sterile PBS, and washed twice. Bacteria (1 ml suspension, 1×10^6^ cell concentrations) were then stained with CFDA, using stock solution (0.5 mM), incubated with shaking for 30 min in 37°C, and washed twice following incubation.

### Identification of rPtsA-Binding Phages

To identify putative rPtsA-binding sequences, we screened a combinatorial peptide library expressed in filamentous phage fth1, as described previously [46]. In short, the type 88 phage display library, in which a second recombinant *pVIII* gene has been incorporated into the fd bacteriophage genome, was used in this study. The recombinant *pVIII* gene also carries a tetracycline resistance gene *tetA*. The particular library used in this study contains a random ~12 amino acid sequence flanked by two constant cysteine residues, thus generating random 12-mer loops expressed on PVIII proteins of a mosaic fd phage. Selection and characterization of the phages were accomplished via tetracycline-resistant bacterial colonies rather than isolating phages from plaques. This library contains 10^8^−10^9^ random inserts in the original phage display library.

After incubation of the Fd phage library on HAT-rPtsA-adsorbed culture dishes, unbound phages were washed out, and bound phages were eluted at pH 2.2. Phages were then retested for their ability to bind rPtsA by using dot blot analysis. The phages were adsorbed onto filter paper, and rPtsA was added, followed by anti-rPtsA antiserum. These dot blots were repeated twice with the phages that demonstrated binding to rPtsA. As a negative control, we used a phage without an insert. The nine identified rPtsA-binding phages were tested for their ability to interfere with bacterial adhesion to cultured A549 cells. To prevent redundancy, we present only the 6 positive phages and the phage without insert. The results with the phages with insert that did not inhibit *S*. *pneumoniae* adhesion to the human cell lines were identical to the results with the phage without insert.

### Bioinformatics Analysis

PtsA sequences were analyzed for homology to human or pneumococcal sequences using the NCBI database. The nucleotide sequences of the peptide insert in rPtsA-binding phages were converted into amino acid sequences and compared to human proteins in UniProt using NCBI's blastp program, with the option "Automatically adjust parameters for short input sequences". Candidate proteins from the blastp results were manually selected according to their membrane or extracellular matrix localization.

### Peptide Synthesis

The following peptides were synthesized according to the sequences in the human protein homologous to the insert peptides in the phages: BMPER (VLVKNDARRTRS), Col VII (VVVSDATRVRVA), MMRN1 (TEQVSDQKNAPA), PCDH19 (DGGVPML), Intβ4 (DQVARIPVIRRV) and Eps 1 (SDGGVPV) and the negative peptide (LPADWATTLMVCSSK) (Hi-Lab laboratories, Rehovot, Israel). Contactin 4 (Q8IWV2 UniProt)-derived peptide (WECKANGRPKPTY) is a putative target for NOX, an adhesin of *S*. *pneumoniae* [17], and was used as a negative control for peptide-rPtsA binding.

### MicroScale Thermophoresis (MST) Analysis

rPtsA labeled with fluorescent NT-495 dye at a constant concentration of 335 nM was mixed with sixteen serial dilutions (~90 nM–3.175 mM) of peptides derived from PCDH19, MMRN1, Eps1, Int β and BMPER. Contactin 4, was used as a negative control [17]. MST analysis was performed using Monolith NT.115 (NanoTemper Technologies GmbH, München, Germany). The normalized change in the fluorescence of bound and unbound labeled rPtsA (ΔF) is indicative of the peptide binding. Plotting ΔF vs. peptide concentration facilitated the generation the dissociation curves, computed by the NTP program. In this way the Kd, reflecting the affinity of each of the peptides for rPtsA, was obtained. The curves obtained from the interaction of rPtsA with its putative target-receptor-derived peptides exhibited a signal-to-noise ratio (SNR) of at least 2, indicating the specificity of the interaction. A curve could not be generated for the control peptide (contactin 4) due to a SNR of less than 1. A representative experiment shown is out of 3 performed. Each experiment was performed in triplicate.

### Human Sera

Sera were collected at 24 and 38 months of age from healthy Bedouin children visiting maternal and child health care (MCHC) clinics for routine checkups. The children were enrolled as controls for an immunization clinical trial between November 2001 and July 2007. For each patient venous blood samples and nasopharyngeal swab were taken (S2 Table). Serum samples were stored at -70°C.

### Ex Vivo Neutralization of *S*. *pneumoniae*

Strain WU2 cells were pretreated with Eps 1, BMPER and PCDH19 peptides and then inoculated IN into adult CBA/N^*xid*^ mice. Mice were euthanized 3, 24 or 48 h later. The nasopharynx and lungs were excised, homogenized and plated onto blood agar plates for counting. As a control, another group of mice was injected with bacteria treated *ex vivo* for 1 h with PBS alone.

In addition, CBA/N^*xid*^ mice were immunized with rPtsA as described above. Mice were euthanized, and blood was drawn from the hearts. WU2 cells were incubated at 37°C for 1 h with 1:20 diluted mice pre-immune or anti-rPtsA antiserum. A lethal dose (10^4^ CFU) of WU2 was inoculated intravenously (IV) into BALB/c or CBA/N^*xid*^ mice. Survival was monitored continuously.

### Statistical Analysis

Non-parametric Pearson correlation analysis (r value) was used to evaluate the dose-dependent inhibition of pneumococci binding to A549 or Detroit 562 cells. To determine the significance of inhibition among all tested groups and relative to the positive control ANOVA analysis and a two-tailed unpaired Student's *t-*test were performed, respectively. Inhibition of bacterial colonization was determined using Student's *t-*test. Analysis of survival was done using the log-rank (Mantel Cox) test Or Gehan-Breslow_Wilcoxon tests using GraphPad Prism V6 software.

## Results

### Cell-Wall Localization and Immunogenicity of PtsA

Our previous 2D PAGE immunoblot analysis studies of the different cell-wall fractions with sera obtained from mice immunized with the total cell-wall lectin or cell-wall non-lectin fractions revealed a highly immunogenic spot in the total and non-lectin fractions (Spot 1) [39]. The spot was found to contain DnaK, which has low antigenicity in humans and is incapable of eliciting a protective immune response in mice [47]. We therefore continued to explore the non-lectin fraction of strain WU2 by MALDI-TOF analysis and found that co-localized with DnaK in Spot 1 was an additional protein, PtsA (PI 4.6, molecular weight 63.4, Mascot score 134, SP_RS05795, WP_000138135). We thus assumed that the immunogenic protein in this spot was PtsA (and not DnaK), an assumption that we confirmed in this study. MALDI-TOF analysis detected PtsA in the cell-wall of seven of the nine different *S*. *pneumoniae* strains tested (S1 Table). Pneumolysin, enolase, and glyceraldehyde 3-phosphate dehydrogenase were identified in the cell-wall preparations of all strains analyzed. These results encouraged us to further explore the function of PtsA in the bacterial cell-wall and its potential as a vaccine.

### Surface Expression of PtsA in Different Pneumococcal Strains

The *pts*A gene was cloned, expressed, and purified, and antibodies were produced as described in the Materials and Methods section (S1 Fig). The surface expression of PtsA was analyzed using rabbit anti-rPtsA antiserum. PtsA was found in the cell-wall, membrane and cytoplasmic cellular fractions of strain WU2 (Fig 1A). Pneumolysin, which is known to be present in the *S*. *pneumoniae* cell wall [26], was used as a positive control for cell-wall localization, and FabD, a protein involved in lipid synthesis, was used as a negative control [27]. Pneumolysin (Fig 1B) was detected in the cell-wall, membrane and cytoplasm of strain WU2, while there was no detection of FabD protein in the bacterial cell-wall (Fig 1C).

To validate the presence of PtsA on the bacterial surface, flow cytometry analysis of live strain R6 probed with a mouse monoclonal anti-rPtsA antibody was undertaken. PtsA was indeed found to be expressed on the bacterial surface (Fig 1D). Purified mouse IgG served as a negative control. Consistently, SIM orthogonal view imaging of live bacteria stained with mouse anti-rPtsA antisera (green) indicated that PtsA (green) was located on the perimeter, surrounding the bacterial genome (white). This configuration was most prominently observed in the XZ plane and further confirmed in the XY and YZ planes (Fig 1E). No such staining was observed with preimmune serum, used as the control.

### PtsA Mediates *S*. *pneumoniae* Adhesion to Target Host Cells

To further establish the extent to which PtsA is involved in pneumococcal interaction with the host, the ability of rPtsA to inhibit pneumococcal adhesion to human lung adenocarcinoma A549 cells was tested using the previously described assay for inhibition of *S*. *pneumoniae* adhesion to A549 cells [16, 17, 41, 48]. rPtsA significantly inhibited the adhesion of the four strains tested: *S*. *pneumoniae* strain WU2 (Fig 2A; p < 0.0001; r = −0.943), the unencapsulated derivative of strain WU2, namely, strain 3.8DW (Fig 2B; p < 0.0001; r = −0.371); serotype 2 strain D39 (Fig 2C; p < 0.0001; r = −1); and the unencapsulated derivative of strain D39, namely, strain R6 (Fig 2D; p < 0.0001; r = −1). All groups demonstrated dose dependency except strain 3.8DW. Nonetheless, a significant reduction, by an order of magnitude, in the adhesion of strain 3.8DW was observed in the presence of rPtsA. KLH was used as a negative control. A representative experiment demonstrating the inability of KLH to inhibit D39 adhesion to A549 cells is presented in Fig 2E (p = 0.8; r = −0.2). Of note, in a previous study [16] no inhibition of adhesion could be observed using either rPsipD, a surface *S*. *pneumoniae* protein not involved in adhesion, or KLH. In the current study we used only KLH as a negative control protein.

We then tested the ability of anti-rPtsA antiserum to inhibit *S*. *pneumoniae* adhesion to A549 cells. *S*. *pneumoniae* cells were incubated with rabbit anti-rPtsA antiserum and then added to the gelatin-blocked A549 cells. Rabbit anti-rPtsA antiserum significantly inhibited the adhesion to A549 cells of strains: WU2 (Fig 2F; p < 0.0001; r = −0.359), 3.8DW (Fig 2G; p < 0.0001; r = −0.886), D39 (Fig 2H; p < 0.0001; r = −0.943) and R6 (Fig 2I; p < 0.0001; r = −0.406). Serum obtained from a rabbit prior to immunization did not inhibit the adhesion of strain WU2 to A549 (Fig 2J; p > 0.005; r = +0.667).

### Identification of rPtsA-Binding Sequences

To identify PtsA target molecules in the host, we screened a combinatorial peptide library expressed in a filamentous phage with rPtsA. Of the nine phages that bound to rPtsA, six inhibited adhesion of strain WU2 to the A549 cells: phage D3 (S2A Fig; p < 0.0001, r = −1); phage E6 (S2B Fig; p < 0.0001, r = −0.6); phage D8 (S2C Fig; p<0.0001, r = −0.8); phage D10 (S2D Fig; p < 0.0001, r = −0.8); phage H9 (S2E Fig; p<0.0001, r = −0.7); and phage H10 (S2F Fig; p < 0.0001 r = −0.164). The phages significantly inhibited the adhesion by more than 75% in comparison to adhesion of bacteria without inhibitors. Moreover, for phage D3, phage D8 and phage D10, the inhibition of adhesion was dose dependent. Inhibition of bacteria below 25% was considered as negative. To prevent redundancy, the controls include the phage without an insert and only one phage with a non-inhibitory insert. The phage without an insert inhibited pneumococcal adhesion to A549 cells only by 20% (S2G Fig p<0.001, r = − 0.7). In addition an inactive page with insert demonstrated only about 15% reduction in bacterial adhesion (S2H Fig; p<0.0001, r = −0.7)."

Sequence determination of the six inhibitory peptide inserts was achieved following sequencing the oligonucleotide inserts in the envelop gene of the phages and translating them into amino acids. The peptide sequences were compared to the UniProt human proteins database by using NCBI's blastp program. Candidate proteins from the blastp results were selected manually according to their localization in the cell membrane or the ECM (Table 1). The following proteins were selected for further study: epsin 1 (Eps 1), bone morphogenetic protein binding endothelial regulator (BMPER), multimerin 1 (MMRN1), protocadherin 19 (PCDH 19), integrin β4 (Int β4) and collagen VII α1 (Col VIIα).

**Table 1:** Inhibitory peptide sequences.

Sequence origin	Peptide Sequence	Acronym	E-value*	% Identity*
**(Phage D3) GCTGTGGTTGTTAATGATGCTTAGCGGACTCGTAAG.**	AVVVNDANRTRK			
**(Phage D8) GCGGATGATGAGAGTCCTGAGATGAAGCCGGGTTAG**	ADDESPEMKPGX			
**(Phage E6) ACGTAGCAGGTGAGTAATCATAAGAATCATACTAAT**	TXQVSNHKNHTN			
**(Phage H9) TTGGATAGTATTCGCATCATCGTAATTCGTCGAAT**	LDSIRIIVIRRI			
**(Phage H10) GCGGATGGGGGGGTGCCGGTT**	ADGGVPV			
**(Phage D10) GATCAGAGGCCTGCGTAGCGGCGGATGTATAGGGCG**	DQRPAXRRMYRA			
(BLAST Result for D3) BMPER protein (Q8N8U9 UniProt)	VLVKNDARRTRS	BMPER**	16	73
(BLAST Result for D3) collagen, type VII, alpha 1 (Q02388 UniProt)	VVVSDATRVRVA	Col VII**	3.1	70
(BLAST results for E6)human multimerin 1 (Q4W5L1 Uniprot)	TEQVSDQKNAPA	MMRN1	4.3	67
(BLAST Result for H10) protocadherin 19 (Q8TAB3 UniProt)	DGGVPML	PCDH19	56	100
(BLAST Result for H9) integrin, beta 4 (P16144 UniProt)	DQVARIPVIRRV	Intβ4	16	75
(BLAST Result for H10) epsin 1 (Q9Y6I3 UniProt)	SDGGVPV (354–360)	Eps 1	6.7	100

* E-value and % identity from Blastp search vs. UniProt.

** The shaded amino acids in BMPER and Col VII derived peptide sequences have 5 and 3 identical and similar amino acids, respectively (LALIGN)

### Existence of the Putative Target Molecules in A549 Cells

Immunofluorescence microscopy revealed the expression in A549 cells of Eps 1, Int β4, BMPER, MMRN1, and PCDH 19, but not of Col VIIα (Fig 3A–3D, respectively). Similar results were obtained for the pharyngeal carcinoma cell line, Detroit 562 (data not shown). Experiments with bacteria stained green with carboxyfluorescein diacetate (CFDA) and A549 cells confirmed the attachment of *S*. *pneumoniae* to the A549 cells (Fig 4A, 4C, 4E and 4G). The interaction of the bacteria with the A549 cells was more clearly visible in Nomarsky microscopy overlaid with the CFDA-stained bacteria (Fig 4B, 4D, 4F and 4H). Furthermore, Fig 4A and 4C imply the association of Int β4 and BMPER with the adhered bacterium. SIM imaging further processed with a maximum intensity projection method revealed that *S*. *pneumoniae* cells (stained with CFDA) adhering to A549 epithelial cells are surrounded by Int β4 (Fig 5A). The higher magnification better demonstrates the bacteria enveloped with Int β4 (Fig 5B), as was further confirmed by the intensity alignment profile (Fig 5C). In addition, *S*. *pneumoniae* cells adhering to A549 cells are shown to be associated with a pedestal like structure. The bacterium 'sits' on a cellular protrusion that contains Int β4 (Fig 5D). The higher magnification provides a better visualization of this association of *S*. *pneumoniae* with the A549 cell via Int β4 in the pedestal-like structure (Fig 5E), as was also confirmed by the intensity alignment profile in Fig 5F. Of note, the interaction of *S*. *pneumoniae* with its target cells consists of sequential stages initiated by pilus binding, in pili-carrying strains, followed by intimate adhesion with different adhesins and culminating in cell invasion with invasins. Two different stages of *S*. *pneumoniae* interaction with its host cell are depicted in Fig 5. The first is attachment to a pedestal-like host-cell structure, and the second is engulfment by the host cell.

### Affinity of rPtsA Interactions with Target-Molecule-Derived Peptides

Following the verification of the existence of the putative receptors for PtsA in the A549 (Figs 3, 4 and 5) and Detroit 562 (data not shown) cells, peptides spanning the regions in the putative target molecules that were homologous to the insert peptides in the inhibitory phages were synthesized (Table 1). MicroScale thermophoresis (MST) was used to test the affinity of rPtsA to the target-derived peptides. Normalized change in the fluorescence of labeled rPtsA following peptide binding facilitated the determination of the affinity of rPtsA for each of the five peptides but not for a control peptide (contactin 4, a peptide derived from the NOX putative receptor [17]), as reflected by their Kd values (Fig 6). Represented here are the affinities for rPtsA binding to: the Eps1-derived peptide Kd = 43.900 ± 9.35 μM; the BMPER-derived peptide—Kd = 21.00± 5.49μM; the MMRN1-derived peptide Kd = 391 ± 72.7 μM; the PCDH19-derived peptide Kd = 58.00 ± 13.4 μM; and the Int β4-derived peptide Kd = 115±20.5μM.

### Target-Derived Peptides Inhibit *S*. *pneumoniae* Adhesion to Epithelial Cells

Peptides spanning the homologous regions in the target molecules were synthesized (Table 1) and tested for their ability to inhibit bacterial adhesion to A549 cells. Peptides derived from Eps 1 significantly inhibited the adhesion to A549 cells of strains WU2 (7A; p < 0.0001, r = −0.943), 3.8DW (7B; p < 0.0001, r = −0.5), D39 (Fig 7C; p < 0.0001, r = −0.943) and R6 (Fig 7D; p < 0.0001, r = −0.406). The dose dependency was more pronounced for the encapsulated strains than for the unencapsulated strains. Similarly, there was a significant inhibition of WU2 and 3.8DW adhesion to lung-derived A549 cells in the presence of peptides derived from BMPER (Fig 8A; WU2 p < 0.0001, r = −0.887; Fig 8B; 3.8 DW p < 0.0001, r = −0.883), Col VIIα (Fig 8C; WU2 p < 0.0001, r = −1; Fig 8D; 3.8DW p < 0.0001, r = −0.886), MMRN1 (Fig 8E; WU2 p < 0.0001, r = −1; Fig 8F. 3.8DW p < 0.0001, r = −0.821), PCDH 19 (Fig 8G; WU2 p < 0.0001, r = −0.829; Fig 8H; 3.8DW p < 0.0001, r = −0.754) and Int β4 (Fig 8I; WU2 p < 0.0001 r = 0.058; Fig 8J; 3.8DW p < 0.0001, r = −0.9). Dose dependency was observed in all cases except for inhibition of strain 3.8DW with PCDH 19 and of WU2 strain with Int β4. No significant inhibition was observed for a scrambled peptide (Fig 8K). Similar results were obtained for the nasopharynx-derived cell line, Detroit 562. There was significant inhibition of the adhesion of *S*. *pneumoniae* strain WU2 to Detroit 562 cells in the presence of peptides derived from: BMPER (S3A Fig; p < 0.0001, r = −0.9), PCDH19 (S3B Fig; p < 0.0001 r = −0.829), Int β4 (S3C Fig; p < 0.001 r = −0.562) and Eps 1 (S3D Fig; p < 0.0001, r = −0.771). Moreover, for BMPER and PCDH19, this inhibition was dose dependent.

### Target-Derived Peptides Inhibit Colonization

**Fig 9:**
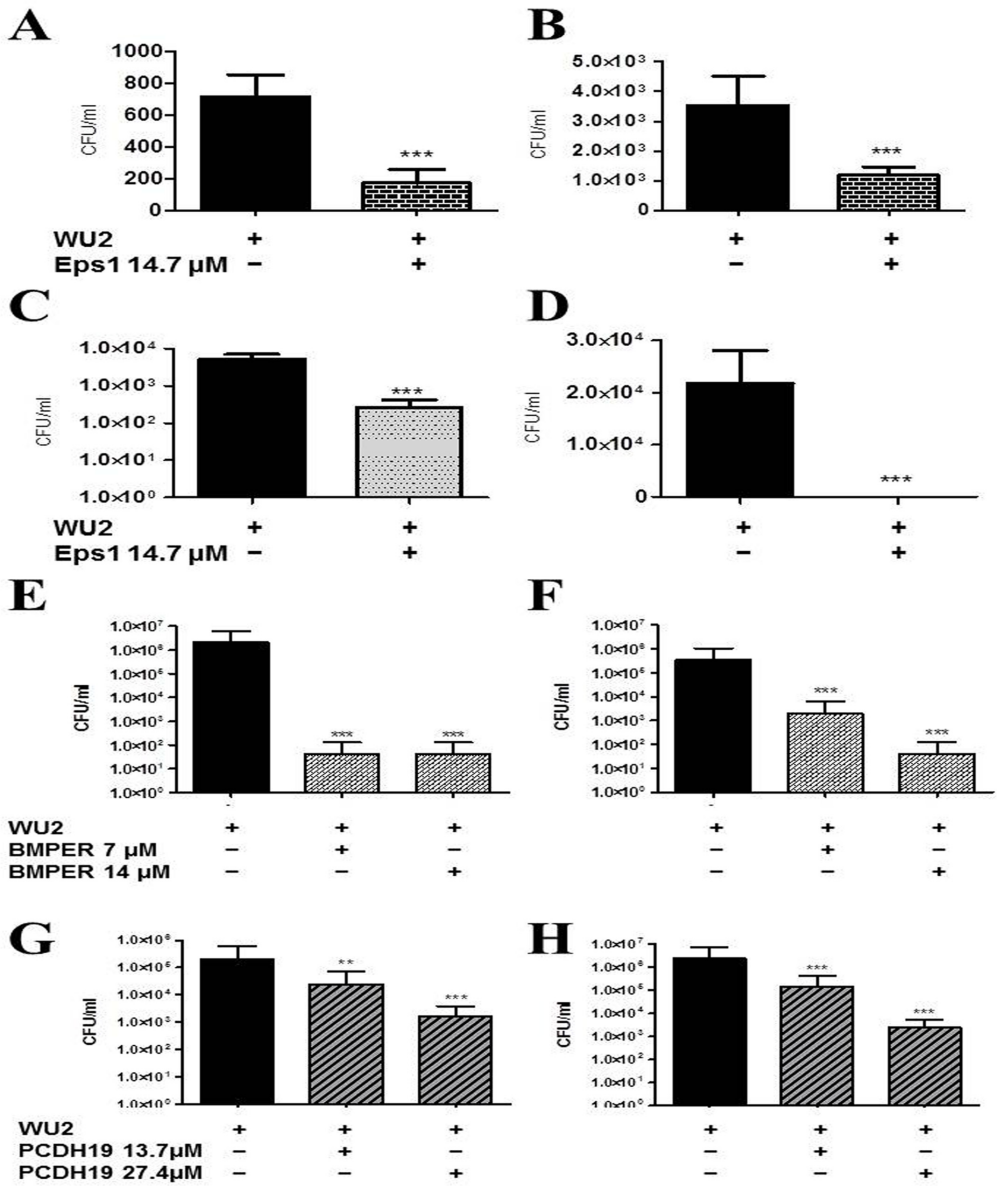
Effects of target-derived peptides on nasopharyngeal and lung colonization and survival in mice. Strain WU2 cells were pretreated with peptide and then inoculated IN into adult CBA/N^*xid*^ mice. As a control, another group of mice was injected with bacteria treated ex vivo for 1 h with PBS alone. Mice were euthanized 3, 24 or 48 h later. The nasopharynx and lungs were excised, homogenized and plated onto blood agar plates for counting. A) Eps 1 (14.7 μM), nasopharynx, 3 h (*P* < 0.001); B) Eps 1 (14.7 μm), lung, 3 h (*P* < 0.0001); C) Eps 1 (14.7 μm), nasopharynx, 24 h (*P* < 0.0001); D) Eps 1 (14.7 μm) lung, 24 h (*P* < 0.0001); E) BMPER, nasopharynx, 48 h at 7 and 14 μM (*P* < 0.0001); F) BMPER, lung, 48 h at 7 and 14 μM (*P* < 0.0001); G) PCDH19, nasopharynx, 48 h at 14.7 and 17.4 μM (*P* < 0.001); H) PCDH19, lung, 48 h (*P* < 0.0001).

The ability of Eps 1 to interfere with bacterial colonization was tested in the intranasal (IN) mouse inoculation model. Strain WU2 bacteria were pretreated with Eps 1 (14.7 μM) and then inoculated IN into CBA/N^*xid*^ mice. The mice were euthanized 3 and 24 h following inoculation, and the nasopharynx and the lungs were excised, homogenized and plated for bacterial colony counting. Mice inoculated with peptide-treated bacteria demonstrated a significant reduction in bacterial load in the nasopharynx and the lungs in comparison to mice inoculated with sham-treated bacteria at 3 h (Fig 9A; *P* < 0.0001; Fig 9B; *P* < 0.0001, respectively) and at 48 h (Fig 9C; *P* < 0.0001) and Fig 9D; *P* < 0.0001) post inoculation. In a different experiment, bacteria were pretreated with BMPER-derived peptide (7 μM and 14 μM) and then inoculated IN into CBA/N^*xid*^ mice. Forty eight hours following inoculation the mice were euthanized, and the nasopharynx and lungs were excised, homogenized and plated onto blood agar plates for bacterial colony counting. A significantly reduced bacterial load was observed in the mice inoculated with BMPER-derived peptide in comparison with mice inoculated with sham-treated bacteria (Fig 9E; *P* < 0.0001 and Fig 9F; *P* < 0.0001, respectively). Similar results were obtained for mice inoculated with WU2 bacteria pretreated with PCDH19-derived peptide (14.7 and 27.4 μM) in comparison with mice inoculated with sham-treated bacteria (Fig 9G; *P* < 0.0001; Fig 9H; *P* < 0.0001).

### Antigenicity of PtsA in Children

Previous studies have revealed that conserved surface proteins demonstrating antigenicity in children constitute immunogens that elicit a protective immune response in mice [47–50]. To test whether PtsA is antigenic in children, rPtsA was immunoblotted with sera obtained from healthy children (S2 Table). PtsA antigenicity was observed in 7 out of 8 children at 24 and 38 months of age (S4 Fig). These results suggest that PtsA is likely to be antigenic in children.

### Vaccine Potential of PtsA

**Fig 10:**
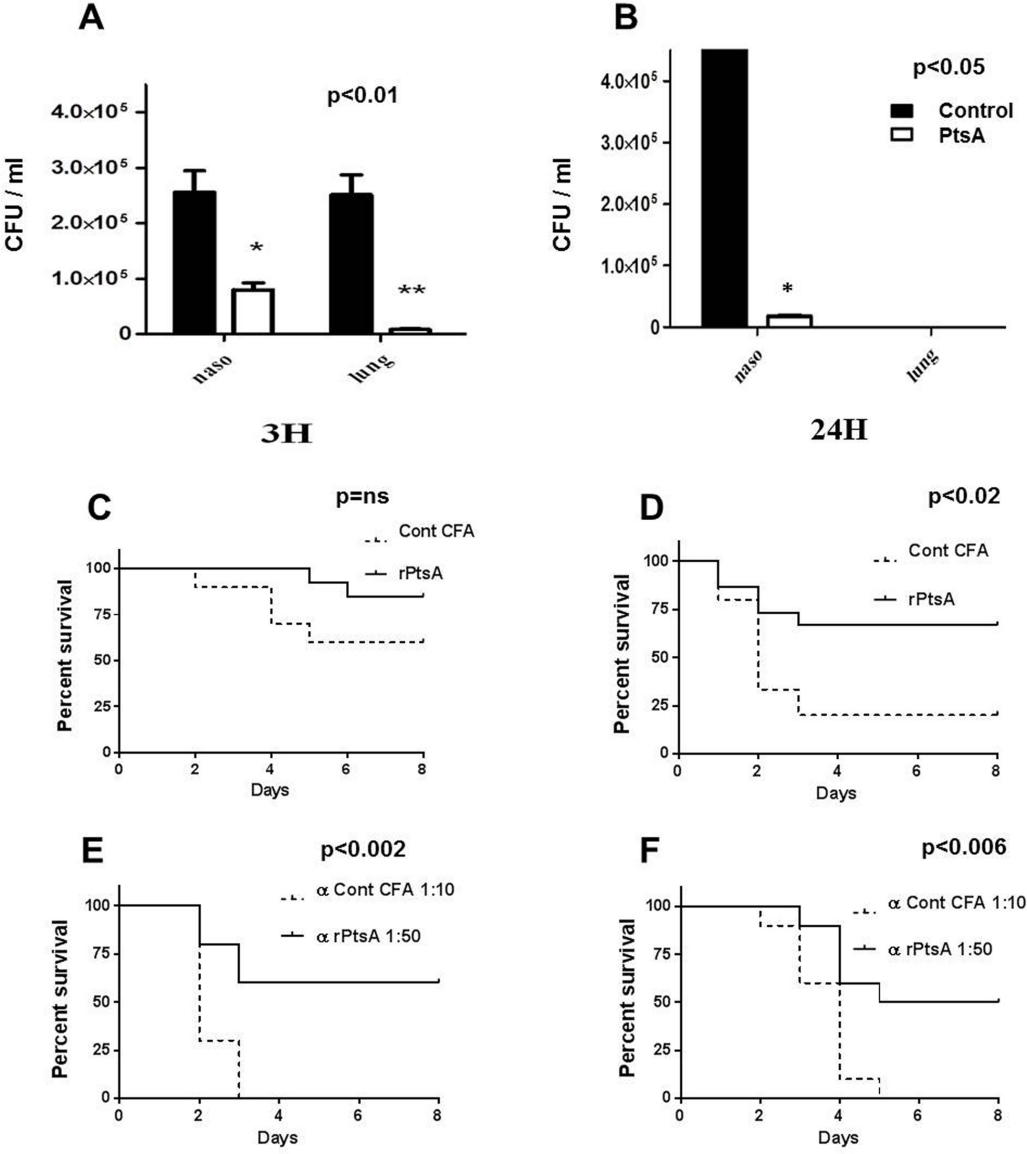
Vaccine potential of rPtsA. BALB/c mice were immunized subcutaneously (SC) with 25 μg of rPtsA emulsified with CFA and boosted (days 14 and 28) with IFA. Mice were challenged IN on day 42 with a sublethal dose (5 × 10^7^) of strain WU2. Mice were sacrificed 3 and 24 h (strain WU2) following inoculation, and the nasopharynx and right lobe lung were excised, homogenized and plated onto blood agar plates for bacterial colony counting. Control mice were immunized with the adjuvant alone. A) Bacterial load in the nasopharynx and the lungs 3 h post inoculation of BALB/c mice with strain WU2 (Student's *t*-test; *P* < 0.01 and *P* < 0.05, respectively). B. Bacterial load in the nasopharynx (*P* < 0.05) and lung of BALB/c mice 24 h post inoculation with WU2 strain. C) After a similar immunization regimen, BALB/c mice were challenged IN with a lethal dose (10^8^ CFU) of strain WU2; control mice were immunized with the adjuvant alone. Mortality was monitored daily. Each group contained 10 mice (p = ns). D) After a similar immunization regimen, CBA/N^*xid*^ mice were challenged intravenously (IV) with a lethal dose (10^4^ CFU) of strain WU2. Control mice were immunized with the adjuvant alone. Mortality was monitored continuously (*P*<0.02). Each group contained 15 mice. E) *S*. *pneumoniae* serotype 3 (10^4^ CFU) was incubated with 1:10 dilution of serum obtained from adjuvant or 1:50 dilution of serum obtained from rPtsA + adjuvant immunized BALB/c mice and inoculated IV into BALB/c mice. Survival was monitored continuously (*P* < 0.002). Each group contained 10 mice. F) *S*. *pneumoniae* serotype 3 strain WU2 was incubated with 1:10 dilution of serum obtained from adjuvant or rPtsA + adjuvant (1:50) immunized CBA/N^*xid*^ mice. Then, 10^4^ CFU of WU2 were inoculated IV into CBA/N^*xid*^ mice, and survival was monitored continuously (*P* < 0.006). Each group contained 10 mice.*Log-rank (Mantel-Cox) test.

The observations that PtsA resides in the cell-wall, is immunogenic, and functions as an adhesin suggest that it could be considered as a candidate for a vaccine antigen. BALB/c mice immunized with rPtsA demonstrated a significant reduction in colonization at 3 h (Fig 10A; p < 0.01) and 24 h (Fig 10B; *P* < 0.05) after inoculation with a sublethal dose of strain WU2. Immunization with rPtsA reduced the mortality of BALB/c mice following an IN inoculation with a lethal dose (10^8^ CFU) of strain WU2, although it did not reach significance (Fig 10C). However, immunization of CBA/N^*xid*^ mice significantly protected the mice against an intravenous (IV) lethal challenge (Fig 10D; *P* < 0.02). In addition, serum obtained from rPtsA-immunized CBA/N^*xid*^ mice neutralized *S*. *pneumoniae* WU2 virulence in BALB/c and CBA/N^*xid*^ mice following IV inoculation with10^4^ CFU of the WU2 bacteria both in BALB/c and CBA/N^*xid*^ (Fig 10E; *P* < 0.002; Fig 10F; *P* > 0.05, respectively). In contrast, serum obtained from CBA/N^*xid*^ mice immunized with adjuvant alone was not able to neutralize bacterial virulence (Fig 10G, Fig 10H, respectively).

## Discussion

Many highly conserved proteins involved in metabolism exhibit a range of additional biological functions important for bacterial virulence [51]. In the cytoplasm of *S*. *pneumonia*, PtsA is the first enzyme of all 21 PTS pathways responsible for the phosphorylation and subsequent import of monosaccharaides as an energy source [31]. The vital role of PtsA in the acquisition of energy sources negates the ability to create null mutant bacteria, just as knockout bacteria cannot be created for other essential proteins and glycolytic enzymes [51]. In this study we found that PtsA is also localized to the cell-wall and the cytoplasmic membrane, where it acts as an adhesin. In a similar manner, it has been found that pneumolysin is present in all these cell fractions, while FabD is localized to the cytoplasmic membrane but not to the cell-wall. Pneumolysin is a cholesterol-dependent cytolysin (CDC) that has long been considered to be a cytoplasmic protein released only upon bacterial autolysis. However, it has recently been found in the cell-wall of strain WU2 [52]. The non-autolytic mechanism of pneumolysin secretion has been shown to be dependent on domain 2 of pneumolysin [53].

In our previous study, in which the same amounts of cell-wall and cytoplasmic proteins were loaded onto the 2D PAGE, a significant difference in the protein distribution was observed. The concentration of each of the identified proteins in the cytoplasm fraction was lower than that in the cell-wall fraction. These results suggest that under our experimental conditions the cell-wall localization of PtsA is not a result of a non-specific leakage from the cytoplasmic to the cell-wall fractions [47]. The protocol for enrichment of cell-wall proteins adapted from Siegel *et al*. demonstrated that no more than 10% of protein leakage from the cytoplasm to the cell-wall fraction can be found [37].

In the current study, enolase, GAPDH and pneumolysin were identified (by MALDI TOF analysis) in the cell-walls of nine different *S*. *pneumoniae* laboratory and clinical isolates, in accordance with previous studies [54, 55]. Importantly, this analysis revealed PtsA in the cell-walls of 7 of these 9 *S*. *pneumoniae* isolates. Further confirmation of PtsA cell-wall localization was obtained by flow cytometry and SIM orthogonal view with anti-rPtsA monoclonal antibodies and polyclonal antisera staining of live bacteria, respectively. The SIM orthogonal view clearly places PtsA at the perimeter, showing that the cell-wall was stained and suggesting that no antibody had penetrated the bacterial membrane. Membrane localization of PtsA observed in the immunoblots may result from its intracellular enzymatic activity in the PTS system, which occurs near or at the inner leaflet of the cytoplasmic membrane. Moreover, bioinformatic analysis predicted the existence of a hydrophobic transmembrane helix region in PtsA (aa 517–535).

The cell-wall localization of PtsA and its immunogenicity encouraged us to further study additional functions of this protein. The hypothetical molecular weight predicted from the NCBI databases for PtsA is 63,165 Da. Following cloning and expression of rPtsA, the molecular weights of tagged-rPtsA and untagged rPtsA were found to be ~75 and ~72 kDa, respectively, possibly as a result of an attached lipid moiety. MALDI-TOF analysis of the untagged protein detected two peaks at 63,932.49 and 62,492.49 Da, values that more closely resemble the predicted molecular weight of the protein.

Infection and spread of *S*. *pneumoniae* is facilitated by the adhesion to host cells that is mediated by surface localized proteins [51, 56, 57]. PtsA may indeed be involved in *S*. *pneumoniae* adhesion to the host, as rPtsA and anti-rPtsA antisera interfered with the adhesion of *S*. *pneumoniae* to human lung adenocarcinoma (A549) cells, in contrast to control proteins rPsipD [16] and rKLH. In the current study, we used the A549 cell line, which has retained the morphological, biochemical and immunological characteristics of type II lung epithelial cells [42–44] and has been widely used as a model to study pneumococcal interaction with human cells [44]. Quantification of *S*. *pneumoniae* adhesion to cultured cells has been performed using various techniques. In some studies, the quantity of fluorescently labeled (FITC or CFDA) *S*. *pneumoniae* per host cell was determined by fluorescent microscopy [14, 18, 19]. In our lab, adhered bacteria are quantified by determining CFU/well, which in our hands is more reproducible and accurate methodology [16, 17].

The bacteria-host interactions facilitating efficient bacterial colonization of the nasopharynx and spread of *S*. *pneumoniae* to the middle ear and the lungs or blood invasion necessitate the development of multiple adhesins, one of which is PtsA. Among the receptors / target molecules to pathogens' adhesins one can find cell adhesion family of molecules [15, 16], other cell-membrane proteins [58, 59] and ECM proteins [60–62]. To identify inhibitors of *S*. *pneumoniae* adhesion and possible PtsA target molecules, a combinatorial peptide library was screened with rPtsA. Six phages inhibited pneumococcal adhesion to A549 cells. The insert peptide sequences found in two of the six phages aligned with Int β4 and PCDH19 cell adhesion molecules. Int β4 has previously been described to be a target for other pathogens [63]. Int β4 may have both direct and indirect effects on pathogenesis during viral and bacterial infections [64, 65]. PCDH19 is a cell adhesion molecule known to control cell movement during neurolation [66], but its association with bacterial infection has not been previously described. Other cell adhesion molecules, such as E-cadherin and flamingo cadherin receptor, have previously been shown to function as receptors for pneumococcal adhesins [15, 16].

Two other proteins carrying homology to the insert peptides in the inhibitory phages were BMPER and Eps 1. BMPER is involved in bone morphogenesis, but it is predicted to be an integral membrane protein as well as a soluble extracellular protein [65], suggesting that it may be involved in bacterial interactions. Eps 1 is an adaptor protein involved in clathrin-coated pit endocytosis or phagocytosis and has also been shown to be used by pathogens as a port of entry to host cells [66–68].

Homologous peptide sequences were also found in the ECM proteins MMRN1 and Col VIIα1. Under natural conditions, the ECM underlies the mucosal epithelial cells. Infection with a pathogen may damage the epithelial cell layer, facilitating exposure of the ECM. Several ECM proteins have been demonstrated to function as microbial targets, including vitronectin [67] and fibronectin [14, 68]. Binding sites for the pathogen in the ECM proteins are different from those for the epithelial cells. Thus, the ECM molecules may function as a bridge between the bacterium and the host cell. MMRN1 is a large, soluble, homopolymeric, factor V binder and a ligand for integrins αIIbβ3 and αvβ3 and can be found in the ECM fibers but not in the plasma. In the cytoplasm, MMRN1 is stored in secretion vacuoles. Collagen is the most abundant family of extracellular proteins. Col VIIα contains many fibronectin type 3 domains and the RGD sequence, which is an integrin ligand [69]. Col VIIα can be found in dermal-epidermal junctions and in mucosal layers [70, 71].

Using immunofluorescence microscopy, we found all the identified proteins in A549 cells, except Col VIIα1. These results may seem contradictory to the inhibitory activity of the Col VIIα-carrying phage on bacterial adhesion to A549 cells. However, this discrepancy can easily be resolved in light of the fact that Col VIIα shares five identical amino acids and three similar amino acids with the BMPER-derived peptide (Table 1).

The adhesion of *S*. *pneumoniae* to the A549 cells was demonstrated by immunofluorescent and Nomarski imaging using CFDA staining. Visible association of CFDA-stained bacteria with Int β4 was shown by immunofluorescence microscopy. Similarly, the association of adhered bacteria to the host cell via BMPER was detected by this method. The Normarski images validated the bacterial adhesion to the cells in all cases, particularly in those with no clear association of the bacteria and the immunostained target molecules.

Interaction of *S*. *pneumoniae* with host cell is probably a multi-stage process. Two stages of pneumococcal interaction with host cells could be visualized using 3D high resolution SIM. In the first stage, the bacterium localizes to a host cell cellular pedestal, and in the second stage the adhered diplococcus is enveloped by Int β4. The signal intensity alignment profile further confirms that Int β4 envelops the bacterium. Additionally, SIM revealed the recruitment of Int β4 to a pedestal-like structure formed by the host cell at the adhesion site during one of the stages of bacterial interaction with the cells. The existence of cellular pseudopodia encircling attached *S*. *pneumoniae* has previously been described by Kimaro Mlacha *et al*. [72]. Pseudopodia and pedestal formation at the site of *E*. *coli* adhesion to epithelial cells have also been previously described [73]. However, this is the first time that the recruitment of Int β4 to a pedestal-like structure that underlies the adhered *S*. *pneumoniae* has been revealed.

All the peptides synthesized according to the sequence in the putative target molecules were found to specifically bind rPtsA (except for the negative control). The affinity binding constant of the rPtsA peptides was in the micromolar range, with the highest to the lowest affinity being BMPER < Eps1 <PCDH19 <Int β4 < MMRN1. As a control we used a peptide (contactin 4-derived) previously shown to bind to another *S*. *pneumoniae* adhesin, NADH oxidase [17].

The Eps 1-derived peptide significantly inhibited the adhesion to A549 of the encapsulated WU2 and D39 strains and of their unencapsulated derivatives 3.8DW and R6. To reduce redundancy, one capsulated strain (WU2) and one unencapsulated (3.8DW) strain were used to test the ability of the other identified target-derived peptides to inhibit the bacterial adhesion. All of the peptides significantly inhibited the adhesion of both bacterial strains to the A549 cells. Most of the peptides inhibited the adhesion in a significant dose dependent manner. Since Col VIIα1 could not be detected in A549 cultures, the unexpected inhibition of bacterial adhesion to A549 cells could have resulted from the high homology it shares with the BMPER-derived peptide (LALIGN).

In addition, the peptides tested (BMPER, PCDH19, Int β4 and Eps 1) significantly inhibited adhesion of strain WU2 to the D562 nasopharyngeal carcinoma-derived cell line, also widely used in studying the interaction of *S*. *pneumoniae* with the host cells [74]. BMPER- and PCDH19-derived peptides significantly inhibited bacterial adhesion in a dose dependent manner. The inhibition of adhesion by Int β4- and Eps 1-derived peptides also showed a dose dependent trend, but the trend was not significant, probably due to the massive reduction of bacterial adhesion at the lowest concentrations of peptides used.

The multitude of putative receptors for each of the different *S*. *pneumoniae* adhesins [11, 13–17, 19–21] is presumably required for effective interaction of the bacterium with organs as different as the lungs [75], the brain [76] and the heart [77]. According to the current study demonstrates that a single target receptor-derived peptide is sufficient for inhibition of the bacterial interaction with the host cells and a massive reduction in bacterial load. These results suggest that some of bacterial interactions formed with the host cells target receptors are interdependent and may occur concomitantly or sequentially, while others may be independent of one other. The effective inhibitory activity of certain target receptor-derived peptides on *S*. *pneumoniae* colonization indicates that those peptides could be suitable candidates for development as therapeutics, as substitutes for or adjuncts to antibiotics, in view of the increasing antibiotic resistance of this pathogen.

Vaccination with the conjugate vaccine and appropriate antibacterial chemotherapy have significantly reduced mortality from pneumococcal diseases [78]. However, *S*. *pneumoniae* presents more than 90 different serotypes, and serotype replacement hampers the efficiency of this vaccine [8, 79]. We were therefore motivated to evaluate the vaccine potential of rPtsA in view of the following properties: rPtsA is a cell-wall immunogenic protein in mice; it is involved in *S*. *pneumoniae* adhesion to the host; it is highly conserved among pneumococcal strains; and it lacks homology to human proteins. In addition, PtsA was found to be antigenic in sera from children. Other antigenic cell surface proteins have previously been shown to elicit protective immune response in mouse models, such as PspA and PspC [19, 80], pneumolysin [81, 82], StkP, PcsB [83], PhtD [84], pilus subunits [85] PsaA [86, 87], FBA, GAPDH, GtS and NADH oxidase [17, 41, 47]. Importantly, we have previously demonstrated that sera drawn from children are capable of inhibiting *S*. *pneumoniae* adhesion to cultured epithelial cells [88].

In the mouse model, immunization with rPtsA reduced nasopharyngeal and lung colonization and partially reduced mortality upon a *S*. *pneumoniae* challenge of both BALB/c and CBA/N^*xid*^ mice, which are mouse strains that are highly resistant and highly susceptible, respectively, to *S*. *pneumoniae* infection. Moreover, mouse anti-rPtsA antiserum, pre-incubated with *S*. *pneumoniae ex vivo*, neutralized bacterial virulence and thereby protected the mice against a lethal challenge with *S*. *pneumoniae*. The presumed mechanism of this neutralization is either by interfering with bacterial adhesion to the host or via enhanced bacterial elimination by phagocytic cells.

## Conclusions

*Streptococcus pneumoniae* is commensal pathogen that upon infection with a virulent strain or co-infection with another pathogen may spread from the nasopharynx or invade the host and cause, among other ailments, otitis media, pneumonia, sepsis and meningitis. Despite the existence of polysaccharide-based vaccines, *S*. *pneumoniae* has remained a major disease-causing pathogen worldwide. To develop new therapies, further understanding of pneumococcal pathogenesis is required. In the current study, we showed a moonlighting protein, phosphoenolpyruvate protein phosphotransferase (PtsA), to be localized to the bacterial cell-wall. On the bacterial surface, this protein was found to function as an adhesin and to mediate bacterial attachment to lung-derived epithelial host cells. Using molecular biology and bioinformatic approaches, we identified PtsA target molecules. The expression of the identified molecules was visualized by immunofluorescent microscopy on the lung derived epithelial cells. Peptides derived from the target molecules were found to specifically bind PtsA and to significantly reduce both bacterial adhesion to host cells *in vitro* and disease development in the mouse model systems *in vivo*. Finally, in the mouse model, PtsA was found to elicit a protective immune response and to significantly reduce colonization and spread to the lung and hence decreased mortality of the mice following a lethal challenge with the pneumococcus.

## Supporting Information

S1 Fig Cloning and purification of PtsA. *ptsa* from strain WU2 was amplified by PCR and inserted into the *E*. *coli* pHAT expression vector used for the transformation of DHα. Insert DNA was analyzed by PCR in ampicillin-resistant transformants (data not shown). The vector was purified and transformed into *E*. *coli* host expression strain BL21(DE3)pLysS. Bacteria were grown overnight, and expression of recombinant rPtsA was induced by use of 1 mmol/L IPTG. The cells were harvested and lysed, and the protein was purified under native conditions and then dialyzed against PBS. The identity of the cloned gene was verified by PCR and by plasmid insert sequencing. (A) SDS-PAGE of HAT-tagged rPtsA protein purified by Ni-affinity chromatography from *E*. *coli* BL21 transformed with the pHAT^*ptsa*^ vector. Lane 1: Molecular weight markers. Lane 2: Coomassie brilliant blue staining of purified rPtsA protein resolved on SDS PAGE. (B) Immunoblotting of the purified PtsA protein with anti-HAT antibodies. Lane 1: Immunoblotting with pre-immune serum. Lane 2: Immunoblotting with anti-HAT antiserum. (C) Coomassie brilliant blue staining of untagged rPtsA separated on SDS PAGE. (D) An immunoblot of untagged rPtsA probed with rabbit anti HAT tagged rPtsA antiserum. (E). MALDI TOF analysis of the untagged rPtsA.
(TIF)

S2 Fig Identification of rPtsA binding sequences. A combinatorial peptide library was screened with rPtsA. The phages that bound rPtsA were tested for their ability to inhibit *S*. *pneumoniae* adhesion to A549 cells. These phages were incubated with strain WU2 for 1 h and added to A549 cells; excess bacteria were removed; and cells were detached with trypsin and plated onto blood agar plates for counting. (A) Phage D3 (p<0.0001, r = -1); (B) Phage E6 (p<0.0001, r = -0.6); (C) Phage D8 p<0.0001, r = -0.8); (D) Phage D10 p<0.0001, r = -0.8); (E) Phage H9 (p<0.0001, r = -0.7); (F) Phage H10 (p<0.0001, r not significant but there was a 75% reduction in adhesion); (G) The phage without an insert did interfere with pneumococcal adhesion to A549 cells, even though it reduced adhesion by only 20% in comparison to ≥ 75% reduction in adhesion in the above active phages (p<0.001 r = -0.7). (H) An inactive phage with an insert demonstrated about 15% reduction in bacterial adhesion (p<0.0001, r = -0.7). Experiments were performed in 3–6 replicates and repeated at least 3 times. Values are means±SD. *Student's *t-*test p<0.05.
(TIF)

S3 Fig Inhibition of adhesion to Detroit 562 cells with target-derived peptides. *S*. *pneumoniae* cells (WU2 strain) were treated for 1 h with each peptide and added to Detroit 562 cells for 2 h; non-adherent bacteria were removed, and cells were detached with trypsin and plated onto blood agar plates for bacterial colony counting. (A) BMPER (p < 0.0001; r = −0.09); (B) PCDH19 (p < 0.0001; r = −0.829); (C) Int β4 (p < 0.0001; r = no dose dependency but 75% inhibition of bacterial adhesion); (D) Eps 1 (p <0.0001; r = −0.771). Experiments were performed in 3–6 replicates and repeated at least 3 times. Values are means±SD. *Student's *t*-test p<0.05.
(TIF)

S4 Fig Antibodies to PtsA present in sera obtained from children. rPtsA was immunoblotted with sera obtained from healthy infants attending day care centers at age: (A) 24 months; (B) 38 months.
(TIF)

S1 Table MALDI_TOF analysis of cell-wall proteins derived of 9 *S*. *pneumoniae* strains. *S*. *pneumoniae* clinical isolates from serotypes 1, 5, 6B, 9V, 14DW, 14R, 23F and laboratory strains from serotypes 2 (D39) and 3 (WU2) were used. Cell wall fractions were isolated using mutanolysin. The cell walls proteins were isolated by 2D PAGE. Protein spots were excised from the gel and subjected to MALDI-TOF-MS analysis
(DOC)

S2 Table Background information for the healthy children. To test whether PtsA is antigenic in children, rPtsA was immunoblotted with sera obtained from healthy children. These healthy children served as control for a Pneumovax clinical trial from 2001–2007.
(DOCX)
