# Supplementary figures and images for: Streptococcus pneumoniae Cell-Wall-Localized Phosphoenolpyruvate Protein Phosphotransferase Can Function as an Adhesin: Identification of Its Host Target Molecules and Evaluation of Its Potential as a Vaccine
Source: PLoS One. 2016 Mar 18;11(3):e0150320. doi: 10.1371/journal.pone.0150320 (PMC4798226; doi:10.1371/journal.pone.0150320)

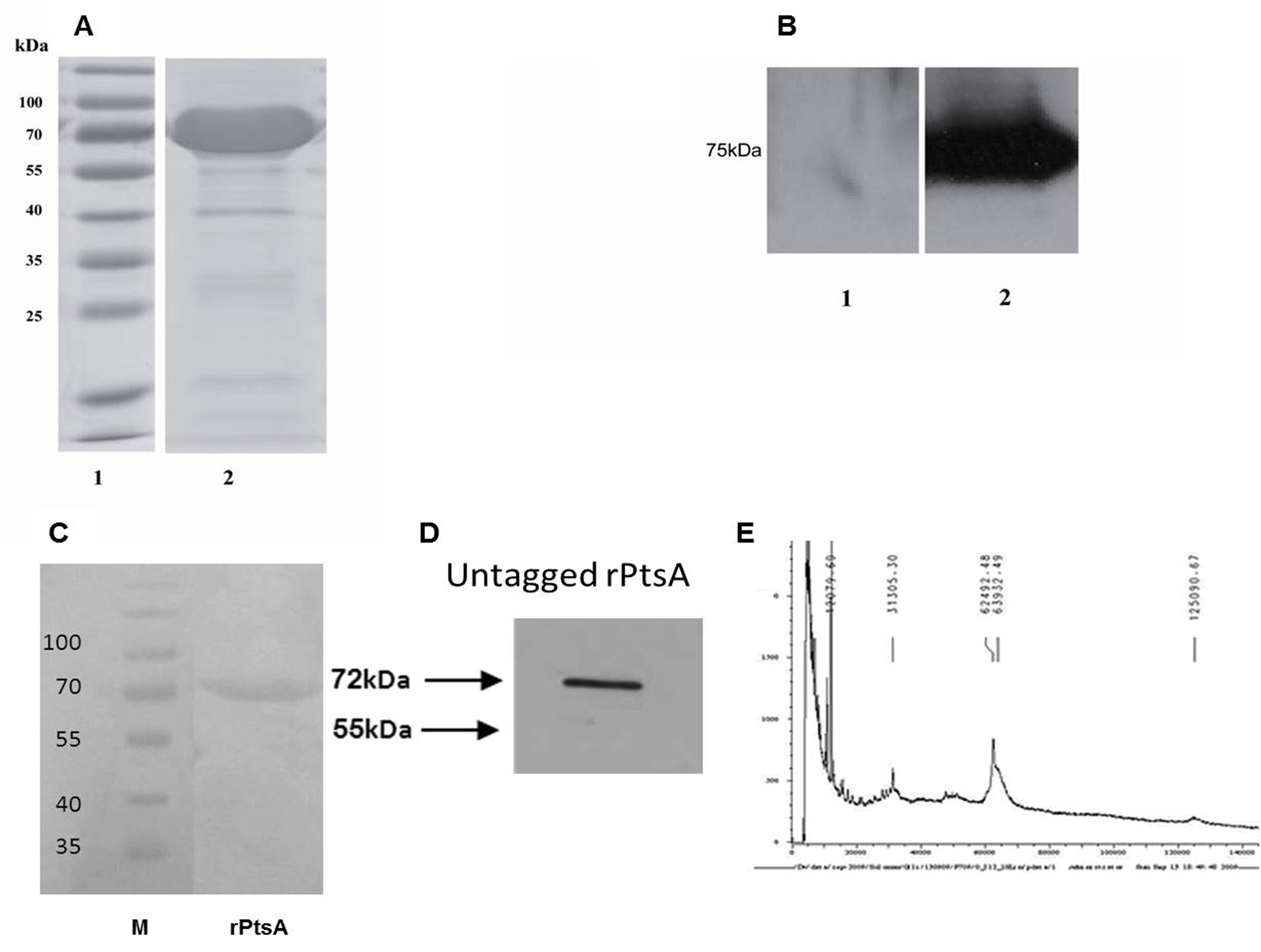

Supplement: S1 Fig — ptsa from strain WU2 was amplified by PCR and inserted into the E. coli pHAT expression vector used for the transformation of DHα. Insert DNA was analyzed by PCR in ampicillin-resistant transformants (data not shown). The vector was purified and transformed into E. coli host expression strain BL21(DE3)pLysS. Bacteria were grown overnight, and expression of recombinant rPtsA was induced by use of 1 mmol/L IPTG. The cells were harvested and lysed, and the protein was purified under native conditions and then dialyzed against PBS. The identity of the cloned gene was verified by PCR and by plasmid insert sequencing. (A) SDS-PAGE of HAT-tagged rPtsA protein purified by Ni-affinity chromatography from E. coli BL21 transformed with the pHATptsa vector. Lane 1: Molecular weight markers. Lane 2: Coomassie brilliant blue staining of purified rPtsA protein resolved on SDS PAGE. (B) Immunoblotting of the purified PtsA protein with anti-HAT antibodies. Lane 1: Immunoblotting with pre-immune serum. Lane 2: Immunoblotting with anti-HAT antiserum. (C) Coomassie brilliant blue staining of untagged rPtsA separated on SDS PAGE. (D) An immunoblot of untagged rPtsA probed with rabbit anti HAT tagged rPtsA antiserum. (E). MALDI TOF analysis of the untagged rPtsA. (TIF) [file pone.0150320.s001.tif]

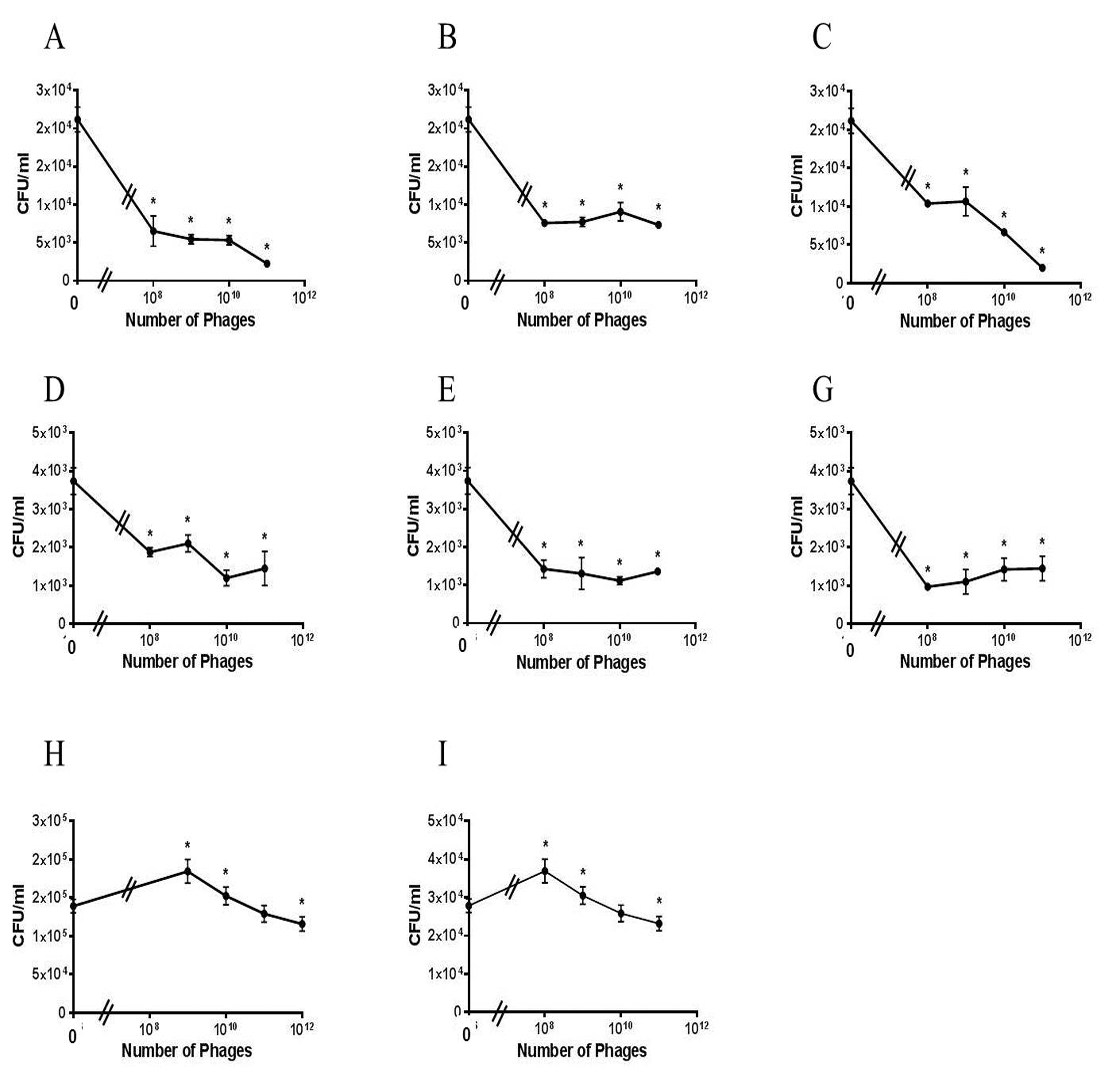

Supplement: S2 Fig — A combinatorial peptide library was screened with rPtsA. The phages that bound rPtsA were tested for their ability to inhibit S. pneumoniae adhesion to A549 cells. These phages were incubated with strain WU2 for 1 h and added to A549 cells; excess bacteria were removed; and cells were detached with trypsin and plated onto blood agar plates for counting. (A) Phage D3 (p<0.0001, r = -1); (B) Phage E6 (p<0.0001, r = -0.6); (C) Phage D8 p<0.0001, r = -0.8); (D) Phage D10 p<0.0001, r = -0.8); (E) Phage H9 (p<0.0001, r = -0.7); (F) Phage H10 (p<0.0001, r not significant but there was a 75% reduction in adhesion); (G) The phage without an insert did interfere with pneumococcal adhesion to A549 cells, even though it reduced adhesion by only 20% in comparison to ≥ 75% reduction in adhesion in the above active phages (p<0.001 r = -0.7). (H) An inactive phage with an insert demonstrated about 15% reduction in bacterial adhesion (p<0.0001, r = -0.7). Experiments were performed in 3–6 replicates and repeated at least 3 times. Values are means±SD. *Student's t-test p<0.05. (TIF) [file pone.0150320.s002.tif]

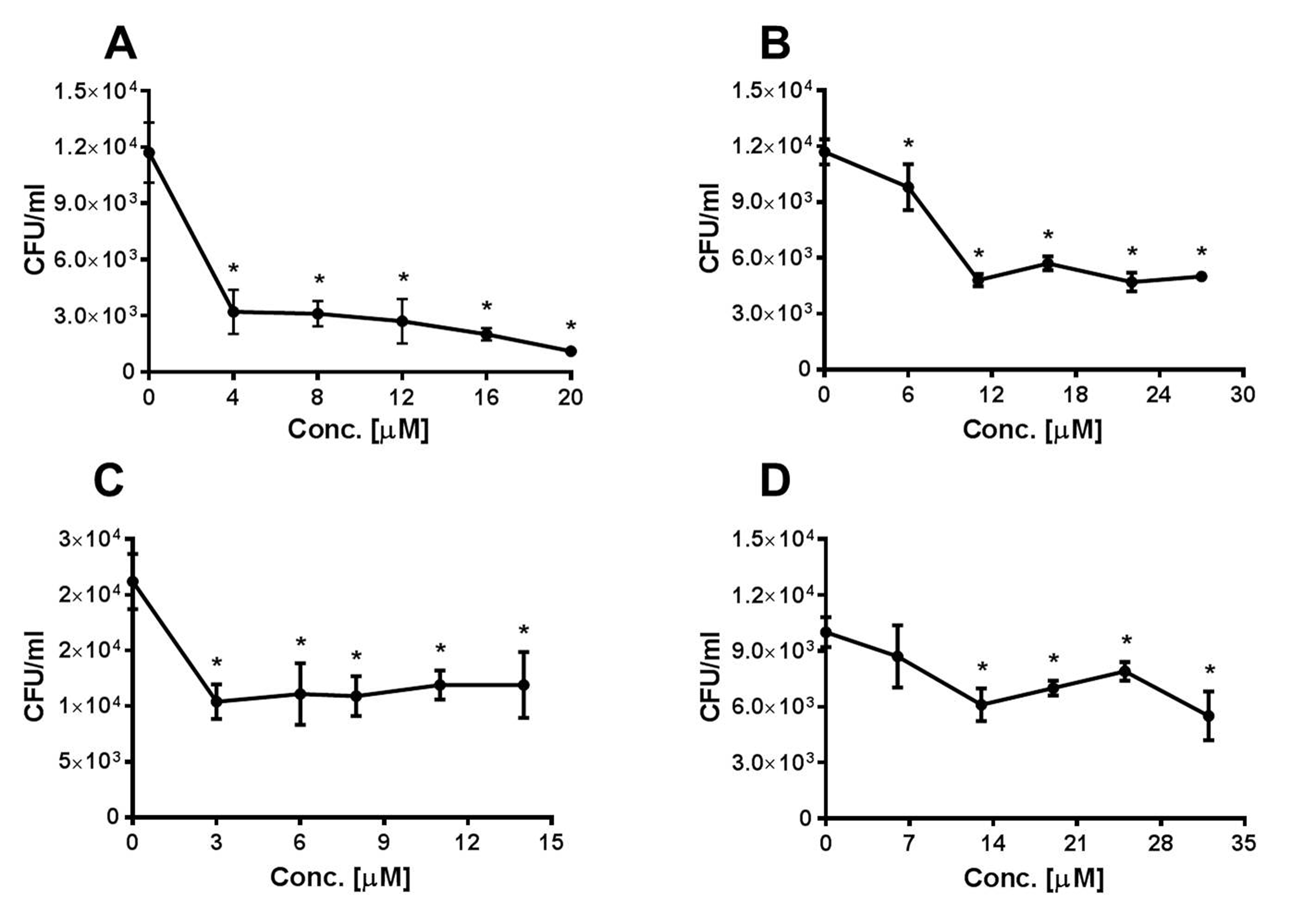

Supplement: S3 Fig — S. pneumoniae cells (WU2 strain) were treated for 1 h with each peptide and added to Detroit 562 cells for 2 h; non-adherent bacteria were removed, and cells were detached with trypsin and plated onto blood agar plates for bacterial colony counting. (A) BMPER (p < 0.0001; r = −0.09); (B) PCDH19 (p < 0.0001; r = −0.829); (C) Int β4 (p < 0.0001; r = no dose dependency but 75% inhibition of bacterial adhesion); (D) Eps 1 (p <0.0001; r = −0.771). Experiments were performed in 3–6 replicates and repeated at least 3 times. Values are means±SD. *Student's t-test p<0.05. (TIF) [file pone.0150320.s003.tif]

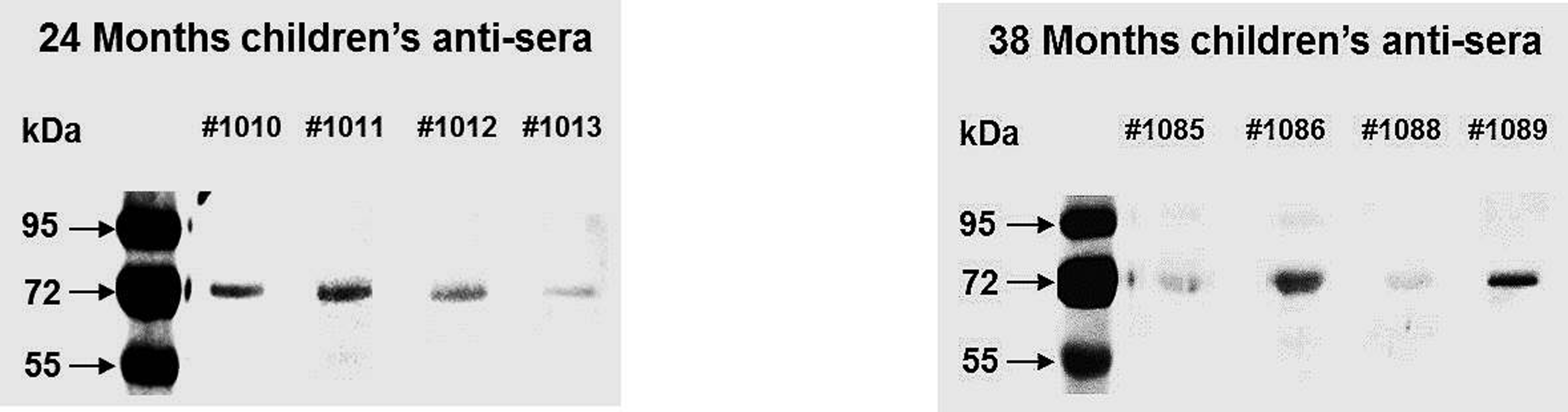

Supplement: S4 Fig — rPtsA was immunoblotted with sera obtained from healthy infants attending day care centers at age: (A) 24 months; (B) 38 months. (TIF) [file pone.0150320.s004.tif]
