# Supplementary material for: Streptococcus pneumoniae Cell-Wall-Localized Phosphoenolpyruvate Protein Phosphotransferase Can Function as an Adhesin: Identification of Its Host Target Molecules and Evaluation of Its Potential as a Vaccine
Source: PLoS One. 2016 Mar 18;11(3):e0150320. doi: 10.1371/journal.pone.0150320 (PMC4798226; doi:10.1371/journal.pone.0150320)
Supplement: S1 Table — S. pneumoniae clinical isolates from serotypes 1, 5, 6B, 9V, 14DW, 14R, 23F and laboratory strains from serotypes 2 (D39) and 3 (WU2) were used. Cell wall fractions were isolated using mutanolysin. The cell walls proteins were isolated by 2D PAGE. Protein spots were excised from the gel and subjected to MALDI-TOF-MS analysis (DOC) [file pone.0150320.s005.doc]

**S1_Table. MALDI_TOF analysis of cell-wall proteins derived of 9 *S. pneumoniae* strains.**

| **Spot** | **Serotype**  **Protein Locus_tag / RefSeq.** | **1** | **2**  **D39** | **3**  **WU2** | **5** | **6B** | **9V** | **14**  **DW** | **14**  **R** | **23F** |
| --- | --- | --- | --- | --- | --- | --- | --- | --- | --- | --- |
| 1 | DNA K SP_RS02530 / WP_000034665.1 | √ | √ | √ | √ | √ | √ | √ | √ | √ |
| 1 | Phosphoenolpyruvate-protein phosphotransferase SP_RS05795 / WP_000138135.1 | √ | √ | √ |  | √ | √ |  | √ | √ |
| 2 | Phosphoglucosamine mutase SP_RS07685 / WP_000521411.1 | √ | √ | √ | √ |  | √ | √ | √ | √ |
| 2 | Endopeptidase O SP_RS08130 / WP_000199265.1 |  |  |  |  |  |  |  |  | √ |
| 3 | Trigger factor SP_RS01985 / WP_000116479.1 | √ | √ | √ | √ | √ | √ | √ | √ | √ |
| 4 | 60kDa chaperonin (GroEL protein) SP_RS09585 / WP_000031573.1 | √ | √ | √ | √ | √ | √ | √ | √ | √ |
| 5 | D-alanine--D-alanine ligase SP_RS08255 / WP_000814631.1 | √ |  | √ | √ | √ | √ | √ | √ | √ |
| 6 | NADH Oxidase SP_RS07220 / WP_000036793.1 | √ | √ | √ | √ | √ | √ | √ | √ | √ |
| 7 | Glutamyl-tRNA Amidotransferase subunit A SP_RS02155 / WP_000143747.1 |  | √ | √ | √ |  |  |  | √ |  |
| 8 | Dipeptidase PepV SP_RS03055 / WP_000125043.1 |  | √ | √ | √ |  | √ |  |  |  |
| 11 | Oligopeptide-binding protein amiA SP_RS09395 / WP_000742235.1 |  | √ | √ |  |  |  |  | √ |  |
| 12 | Pneumolysin (thiol-activated cytolysin) SP_RS09670 / WP_001284361.1 | √ | √ | √ | √ | √ | √ | √ | √ | √ |
| 13 | L-lactate dehydrogenase SP_RS05980 / WP_000204727.1 | √ | √ | √ | √ | √ | √ | √ | √ | √ |
| 14 | Glyceraldehyde-3-phosphate dehydrogenase SP_RS10185 / WP_000260666.1 | √ | √ | √ | √ | √ | √ | √ | √ | √ |
| 15 | Fructose-biphosphate aldolase SP_RS02975 / WP_001019003.1 | √ | √ | √ | √ | √ | √ | √ | √ | √ |
| 16 | UDP-glucose 4-epimerase SP_RS07925 / WP_001156518.1 | √ | √ | √ | √ | √ | √ | √ | √ | √ |
| 17 | Elongation factor G SP_RS01330 / WP_000090344.1 | √ | √ | √ | √ | √ | √ | √ |  | √ |
| 19 | Pyruvate Oxidase SP_RS03575 / WP_000191798.1 | √ | √ | √ | √ | √ | √ | √ | √ | √ |
| 20 | Pyruvate kinase SP_RS04425 / WP_001042809.1 | √ | √ | √ | √ | √ | √ | √ | √ | √ |
| 21 | Elongation factor TU SP_RS07325 / WP_001040724.1 | √ | √ | √ |  | √ | √ | √ | √ | √ |
| 21 | GMP synthase SP_RS07100 / WP_000065723.1 |  |  | √ |  |  | √ |  |  |  |
| 22 | Glutamyl-tRNA synthetase SP_RS10450 / WP_001277935.1 | √ | √ | √ | √ | √ | √ | √ | √ | √ |
| 23 | NADP-specific glutamate dehydrogenase SP_RS06400 / WP_000199560.1 | √ | √ | √ | √ | √ | √ | √ | √ | √ |
| 23 | Ornithine carbamoyltransferase SP_RS10960 / WP_000185363.1 |  |  |  |  |  | √ |  | √ |  |
| 23 | Dihydrolipoyl dehydrogenase SP_RS05725 / WP_001162908.1 |  |  |  |  | √ |  |  |  | √ |
| 25 | Enolase (2-phosphoglycerate dehydratase) SP_RS05590 / WP_000022813.1 | √ | √ | √ | √ | √ | √ | √ | √ | √ |
| 26 | Elongation factor Ts SP_RS11305 / WP_000808063.1 | √ | √ | √ | √ |  |  |  |  |  |
| 27 | Phosphoglycerate kinase SP_RS02455 / WP_001096747.1 | √ | √ | √ | √ | √ | √ | √ | √ | √ |
| 29 | Glucose-6-phosphate isomerase SP_RS10545 / WP_000018264.1 | √ | √ | √ | √ | √ | √ | √ | √ | √ |
| 30 | 6-phosphogluconate dehydrogenase SP_RS01835 / WP_000158781.1 | √ | √ | √ | √ | √ | √ | √ | √ | √ |
| 31 | Aminopeptidase C SP_RS01380 / WP_000991674.1 |  |  | √ |  |  |  |  |  |  |
| 33 | Carbamoyl-phosphate synthase SP_RS06250 / WP_001126406.1 |  |  | √ |  |  |  |  |  |  |
| 33 | Deoxyribose-phosphate aldolase SP_RS04125 / WP_000773677.1 |  | √ | √ |  |  | √ |  |  |  |
| 37 | Penicillin binding protein 1a SP_RS01800 / WP_001040013.1 |  |  |  |  | √ |  |  |  |  |
| 46 | Adenylosuccinate synthetase SP_RS00135 / WP_000205044.1 |  | √ | √ | √ |  |  |  |  |  |
| 49 | Tyrosyl-tRNA synthetase SP_RS10695 / WP_000546887.1 | √ | √ | √ | √ | √ | √ | √ | √ | √ |
| 50 | N-acetylglucosamine-6-phosphate deacetylase SP_RS10395 / WP_001134456.1 |  | √ |  |  |  |  |  |  |  |
| 50 | Serine hydrolase SP_RS00050 / WP_001224760.1 |  | √ |  |  |  |  |  |  |  |
| 52 | Lactate oxidase SP_RS03500 / WP_000120722.1 | √ |  | √ |  |  | √ |  | √ |  |
| 57 | Mannose-specific IIAB components spr0261 / NP_357855.1 |  |  | √ |  |  |  |  |  |  |
| 58 | Ribosomal protein S2 SP_RS11310 / WP_000268465.1 | √ |  | √ | √ |  |  |  |  |  |
| 59 | Manganese-dependent inorganic pyrophosphatase SP_RS07555 / WP_000036043.1 |  | √ | √ | √ |  |  |  | √ |  |
| 60 | Thioredoxin reductase SP_RS07165 / WP_000272304.1 | √ |  | √ | √ |  | √ |  | √ |  |
| 62 | Dihydroorotate dehydrogenase SP_RS03730 /  WP_000255160.1 | √ | √ | √ | √ |  |  |  |  |  |
| 65 | Hypothetical protein spr0491 / NP_358085.1 |  | √ |  |  |  |  |  |  |  |
| 65 | Aspartate carbamoyltransferase SP_RS06260 / WP_001293838.1 |  |  | √ |  |  |  |  |  |  |
| 70 | 1-phosphofructokinase, putative SP_RS04320 /WP_000640786.1 |  | √ | √ |  |  |  |  |  |  |
| 71 | ABC transporter, ATP-binding protein SP_RS02390 / WP_000656548.1 | √ |  | √ |  |  |  |  |  |  |
| 74 | Glucosamine-6-phosphate isomerase SP_RS06935 / WP_000864616.1 |  | √ | √ | √ |  |  |  |  |  |
| 75 | Triosephosphate isomerase SP_RS07760 / WP_000087897.1 | √ | √ | √ | √ |  |  |  | √ |  |
| 76 | Superoxide dismutase SP_RS10385 / WP_000286415.1 | √ | √ | √ |  |  |  |  |  |  |
| 78 | Adenylate kinase SP_RS01115 / WP_001050436.1 |  |  |  |  |  |  |  |  | √ |
| 80 | 2,3-bisphosphoglycerate-dependent phosphoglycerate mutase SP_RS08170 / WP_000240129.1 | √ | √ | √ | √ | √ | √ | √ | √ | √ |
| 84 | Uracil phosphoribosyltransferase SP_RS03650 / WP_000515972.1 | √ |  | √ | √ |  |  |  |  |  |
| 87 | General stress protein SP_RS08955 /  WP_000064115.1 |  | √ |  |  |  |  |  |  |  |
| 105 | Arginine deiminase SP_RS10955 / WP_000094620.1 | √ |  | √ | √ | √ | √ | √ |  | √ |
| 108 | Argininosuccinate synthase SP_RS00575 / WP_033604808.1 |  |  | √ | √ |  |  |  |  |  |
| X1 | Formate-tetrahydrofolate ligase SP_RS06025 / WP_000845295.1 |  | √ | √ |  |  |  |  |  |  |
| X2 | Aminopeptidase N SP_RS03900 / WP_001149111.1 |  |  |  |  | √ |  | √ |  | √ |
| T4 | DNA-directed RNA polymerase, alpha chain SP_RS01140 /  WP_000568988.1 |  | √ |  |  |  |  |  |  |  |
| T5 | Nicotinate phosphoribosyltransferase SP_RS06970 / WP_000283118.1 |  | √ |  |  |  |  |  |  |  |
| T9 | Transketolase SP_RS07965 / WP_000058257.1 |  | √ |  |  |  |  |  |  |  |
