# Supplementary material for: Streptococcus pneumoniae Cell-Wall-Localized Phosphoenolpyruvate Protein Phosphotransferase Can Function as an Adhesin: Identification of Its Host Target Molecules and Evaluation of Its Potential as a Vaccine
Source: PLoS One. 2016 Mar 18;11(3):e0150320. doi: 10.1371/journal.pone.0150320 (PMC4798226; doi:10.1371/journal.pone.0150320)
Supplement: S2 Table — To test whether PtsA is antigenic in children, rPtsA was immunoblotted with sera obtained from healthy children. These healthy children served as control for a Pneumovax clinical trial from 2001–2007. (DOCX) [file pone.0150320.s006.docx]

| Laboratory number | Study^a^ number | Gender | Age (months) | Serotype carried |
| --- | --- | --- | --- | --- |
| 1010 | C0014 | F | 24 | 35B |
| 1011 | C0019 | M | 24 | 21 |
| 1012 | C0318 | F | 24 | 23F |
| 1013 | C0325 | M | 24 | 9V |
| 1085 | C0045 | M | 36 | 15B |
| 1086 | C1664 | M | 36 | 19F |
| 1088 | C0713 | F | 36 | 9V |
| 1089 | C0866 | M | 37 | 19F |

Table S2. Background information for the healthy children

^a^The healthy children served as control for a Pneumovax clinical trial from 2001-2007

^b^Male

^C^Female

^d^No growth

^e^No *S. pneumoniae* growth
